# Functional analysis of *Arabidopsis* immune-related MAPKs uncovers a role for MPK3 as negative regulator of inducible defences

**DOI:** 10.1186/gb-2014-15-6-r87

**Published:** 2014-06-30

**Authors:** Nicolas Frei dit Frey, Ana Victoria Garcia, Jean Bigeard, Rim Zaag, Eduardo Bueso, Marie Garmier, Stéphanie Pateyron, Marie-Ludivine de Tauzia-Moreau, Véronique Brunaud, Sandrine Balzergue, Jean Colcombet, Sébastien Aubourg, Marie-Laure Martin-Magniette, Heribert Hirt

**Affiliations:** 1 Unité de Recherche en Génomique Végétale (URGV), UMR INRA 1165 - Université d’Evry Val d’Essonne - ERL CNRS 8196 - Saclay Plant Sciences, 2 rue Gaston Crémieux, Evry 91057, France; 2 Present address: Laboratoire de Recherche en Sciences Végétales (LRSV), UMR 5546, Université Paul Sabatier/CNRS, 24, chemin de Borde Rouge B.P. 42617 Auzeville, Castanet-Tolosan 31326, France; 3 Institut de Biologie des Plantes (IBP), CNRS-Université Paris-Sud - UMR 8618 - Saclay Plant Sciences, Orsay, Cedex 91405, France; 4 Unité de Recherche en Génomique Végétale (URGV), Plateforme Transcriptome, UMR INRA 1165 - Université d’Evry Val d’Essonne - ERL CNRS 8196, 2 rue Gaston Crémieux, Evry 91057, France; 5 AgroParisTech, UMR 518 MIA, Paris 75005, France; 6 INRA, UMR 518 MIA, Paris 75005, France; 7 Center for Desert Agriculture, 4700 King Abdullah University of Sciences and Technology, Thuwal 23955-6900, Saudi Arabia

## Abstract

**Background:**

Mitogen-activated protein kinases (MAPKs) are key regulators of immune responses in animals and plants. In *Arabidopsis*, perception of microbe-associated molecular patterns (MAMPs) activates the MAPKs MPK3, MPK4 and MPK6. Increasing information depicts the molecular events activated by MAMPs in plants, but the specific and cooperative contributions of the MAPKs in these signalling events are largely unclear.

**Results:**

In this work, we analyse the behaviour of *MPK3*, *MPK4* and *MPK6* mutants in early and late immune responses triggered by the MAMP flg22 from bacterial flagellin. A genome-wide transcriptome analysis reveals that 36% of the flg22-upregulated genes and 68% of the flg22-downregulated genes are affected in at least one *MAPK* mutant. So far MPK4 was considered as a negative regulator of immunity, whereas MPK3 and MPK6 were believed to play partially redundant positive functions in defence. Our work reveals that MPK4 is required for the regulation of approximately 50% of flg22-induced genes and we identify a negative role for MPK3 in regulating defence gene expression, flg22-induced salicylic acid accumulation and disease resistance to *Pseudomonas syringae.* Among the *MAPK*-dependent genes, 27% of flg22-upregulated genes and 76% of flg22-downregulated genes require two or three MAPKs for their regulation. The flg22-induced MAPK activities are differentially regulated in *MPK3* and *MPK6* mutants, both in amplitude and duration, revealing a highly interdependent network.

**Conclusions:**

These data reveal a new set of distinct functions for MPK3, MPK4 and MPK6 and indicate that the plant immune signalling network is choreographed through the interplay of these three interwoven MAPK pathways.

## Background

Plants fend off most microbial attacks thanks to a multi-layered immune system, which is activated through the recognition of diverse microbial features. The first layer of induced defences relies on pattern recognition receptors (PRRs) that detect conserved microbe-associated molecular patterns (MAMPs) and initiate a defence program called pattern-triggered immunity (PTI). All known plant PRRs are located at the plasma membrane where they recognise and bind extracellular MAMPs [1]. The best studied example is FLS2 (flagellin-sensing 2), a receptor kinase with an extracellular leucine-rich repeat (LRR) domain that binds the conserved flg22 epitope derived from bacterial flagellin [2]. This recognition event induces immediate FLS2 association to the co-receptor BAK1 (BRI1-associated kinase 1) and their reciprocal kinase activation, which in turn initiates a series of responses important for defence activation [3]. In plants, MAMP perception induces early and late cellular processes, such as calcium fluxes, kinase cascades, production of reactive oxygen species (ROS), transcriptional reprogramming and reinforcement of the cell wall via deposition of callose [4]. The importance of PTI was highlighted by the identification of pathogen effector molecules that target PTI components to suppress host defences and allow invasion [5]. Through the use of secretion systems, pathogens deliver a suite of effectors to the plant apoplast and intracellular compartments to modify the host cell to their benefit. As a counterpart, plants evolved intracellular receptors with nucleotide-binding and leucine-rich repeat domains (NB-LRR) that sense effectors and activate effector-triggered immunity (ETI) [5]. ETI is an amplified PTI response that results in disease resistance and is often associated with the accumulation of the hormone salicylic acid (SA) and a localised programmed cell death referred to as hypersensitive response (HR). While this response is efficient against biotrophic pathogens, necrotrophic pathogens that kill host cells are fought through activation of defences mediated by the hormones jasmonic acid (JA) and ethylene (ET) [6].

Following the detection of pathogens, MAPK cascades become activated and are central to the regulation of the immune system in animals and plants [7]. These conserved signalling modules are generally composed of a MAPKKK (MAPK kinase kinase), a MAPKK (MAPK kinase) and a MAPK, and function to translate extracellular stimuli into intracellular responses. In plants, MAPKs play important roles in different developmental processes and stress responses, but the far best studied examples are the roles of the MAPKs MPK3, MPK4 and MPK6 in disease resistance [7]. In Arabidopsis, flg22 recognition activates at least two MAPK signalling pathways. One of these MAPK cascades is defined by the MAPKKs MKK4 and MKK5, which act redundantly to activate the MAPKs MPK3 and MPK6 [8]. The second cascade activated by flg22 is defined by the MAPKKK MEKK1, which activates MKK1 and MKK2 that act redundantly on MPK4 [9,10]. It was recently shown that this cascade negatively regulates the MAPKKK MEKK2 (SUMM1) and the NB-LRR SUMM2, whose activation initiates defence responses [11-13]. As a consequence, the double mutant *mkk1 mkk2* and the single mutants *mekk1* and *mpk4* exhibit similar autoimmune phenotypes, such as dwarfism, cell death lesions, ROS accumulation and constitutive SA-mediated defences [9,10,14-16]. Furthermore, *mkk1 mkk2* and *mpk4* plants show enhanced resistance to the biotrophic pathogens *Hyaloperonospora arabidopsidis* and *Pseudomonas syringae* (*P. syringae*) and susceptibility to the necrotrophic fungi *Botrytis cinerea* (*B. cinerea*) (*mkk1 mkk2*) and *Alternaria brassicicola* (*mpk4*) [9,10,16,17]. These phenotypes are partially suppressed by the expression of the bacterial salicylate hydroxylase *NahG* or by mutations that impair SA accumulation [10,15,16]. Recently, the activity of a fourth MAPK, MPK11, was shown to be induced by flg22 and to play redundant functions with the other stress-induced MAPKs in embryo development, but no major function in disease resistance could be detected [18]. Besides induction of the MAPK activities, MAMP treatment also leads to transcript accumulation of *MPK11* and *MPK3* but not of *MPK4* and *MPK6*[19]. The importance of these protein kinases for immunity was further highlighted by the identification of pathogen effectors that target MAPK cascades. *P. syringae* encodes at least two effectors that reduce MAPK activation: the ADP-ribosyltransferase HopF2 that inactivates MKK5 and the phosphothreonine lyase HopAI1 that presumably dephosphorylates MPK3, MPK4 and MPK6 [13,20,21].

While *mpk4* has severe developmental defects, *mpk3* and *mpk6* single mutants resemble wild type plants and only the combination of both mutations impairs normal development. Indeed, MPK3 and MPK6 redundantly regulate stomatal development and the *mpk3 mpk6* double mutant is embryo lethal [22]. MPK3 and MPK6 are believed to be redundant also during plant immune responses, but increasing evidence points to additional independent functions. MPK3 and MPK6 phosphorylate and stabilise the ET biosynthetic enzymes ACS2 and ACS6 and thereby drive ET production in response to *B. cinerea*[23]. Furthermore, both kinases mediate the *B. cinerea-*induced phosphorylation of the transcription factors ERF6 and WRKY33, which in turn regulate defence gene expression and the accumulation of the antimicrobial compound camalexin, respectively [24-26]. In contrast to these redundant functions, MPK3 and MPK6 play different roles in the defence response to *B. cinerea.* While *mpk3* plants are more susceptible to *B. cinerea*, *mpk6* mutants show wild type susceptibility levels and are compromised in the elicitor-induced fungal resistance [25,27]. Furthermore, *mpk6* but not *mpk3* was shown to suppress exacerbated stress responses, as the enhanced resistance of mutants in the phosphatase MKP1 [28], the constitutive stress responses triggered by a dominant allele of the receptor-like wall associated kinase WAK2 (generated by a WAK2-cTAP fusion) [29], or the deregulated cell death triggered by fumonisin B1 [30,31]. MPK3 and MPK6 have also been proposed to play both redundant and distinct roles in the flg22-induced pathway [8,27,32]. Both *mpk3* and *mpk6* single mutants are defective in flg22-induced stomatal closure, a key defence step against pathogen entry into leaves [32]. In contrast, *mpk3* but not *mpk6*, shows increased responses to flg22 in terms of ROS production and growth inhibition [33]. In response to flg22, MPK3 and MPK6 regulate the transcription factors WRKY22 and WRKY29 [8], whereas the ET-related ERF104 is specifically targeted by MPK6 [34]. In agreement with these partially overlapping roles, the use of random peptide libraries and protein arrays suggested common and specific substrates for these immune related MAPKs, among which numerous transcription factors are found [35-37].

All these data indicate that MPK3, MPK4 and MPK6 are key regulators of the transcriptional reprogramming in response to many stresses including MAMP perception. Nevertheless, no transcriptome analysis has been reported that would give insight into the genes controlled by these three MAPKs in response to flg22. Whereas *mpk4* adult plants show strong dwarfism, young *mpk4* seedlings display less severe developmental changes and therefore facilitate the phenotypic analyses of the mutant. In this work, we performed a comparative analysis of MPK3, MPK4 and MPK6 mutants for early (flg22-induced transcriptome changes and MAPK activities) and late (SA production, callose deposition and resistance against *P. syringae*) immune responses. By using a clustering approach based on the transcriptome analysis and a gene network construction method we were able to predict specific transcription factors involved in the flg22-induced transcriptional reprogramming modulated by the individual MAPKs. The analysis of the flg22-induced transcriptomes and the differential regulation of the MAPK activities in the *MAPK* mutants revealed extensive cooperative and inhibitory cross-talk between the MAPK signalling pathways. These analyses also identified new functions for MPK3 and MPK4. Although our and other groups have documented a negative role of MPK4 in the regulation of SA-mediated immunity [16,17,38], our present analysis revealed that MPK4 also functions as a positive regulator of early flg22-induced transcriptional reprogramming. Moreover, MPK3 was found to repress the constitutive and flg22-induced expression of defence genes, inhibit flg22-induced SA accumulation and resistance to *P. syringae*.

## Results

### General overview of the transcriptomes of *mpk3*, *mpk4* and *mpk6* in response to flg22

MPK3, MPK4, MPK6 and recently also MPK11 have been described to be rapidly and transiently activated in response to flg22 and other MAMP treatments [8,9,18]. MAPKs are important regulators of gene transcription in animals [39] and plants [7,36]. To identify genes regulated by the MAPKs in response to flg22, we performed a whole transcriptome analysis of Col-0, *mpk3*, *mpk4* and *mpk6* after mock or 30 min treatment with 1 μM flg22. We took advantage of the root developmental phenotype present in young *mpk4* seedlings to select for homozygous mutant plants from a segregating population (see Material and Methods). The *mpk11* mutant displays only minor and non-reproducible alterations in the flg22-induced transcriptional reprogramming [18] and was therefore not included in the analysis.

#### mpk3 and mpk4 display major and partially overlapping transcriptional changes under standard growth conditions

**Figure 1:**
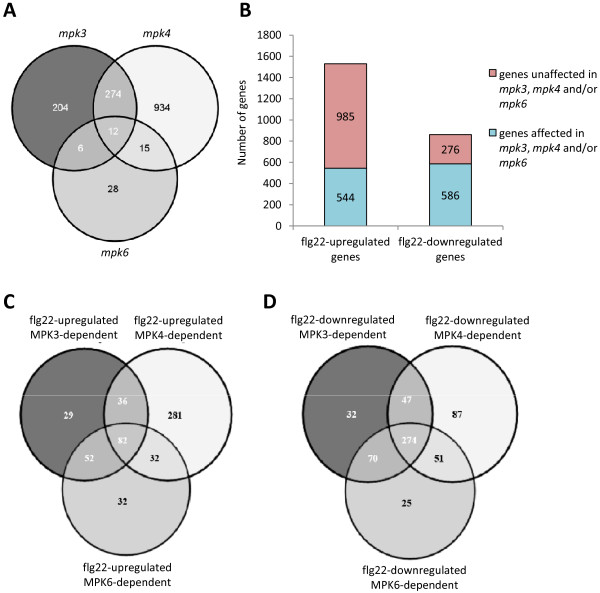
**Transcriptome analysis of *mpk3*, *mpk4 *and *mpk6*. (A)** Venn diagram of the overlap between the differentially expressed genes (up- and downregulated) in control conditions in *mpk3*, *mpk4* and *mpk6* compared to Col-0*.***(B)** Number of genes losing part of their flg22-regulation (at least 1 log ratio) in at least one *MAPK* mutant and number of genes not affected by *MAPK* mutations. **(C)** Venn diagram of the overlap between genes showing flg22-upregulation in Col-0 and affected in *mpk3*, *mpk4* or *mpk6*. **(D)** Venn diagram of the overlap between genes showing flg22-downregulation in Col-0 and affected in *mpk3*, *mpk4* or *mpk6*.

In control conditions, we observed 1,235 genes differentially expressed in *mpk4*, 496 genes in *mpk3* and only 61 genes in *mpk6* in comparison to Col-0 (Table 1, Additional file 1: Table S1 and Additional file 2: Table S2). As the variances of the expression differences were of the same order, we concluded that the transcriptome differences were not due to any experimental error and reflected the impact of the mutations. The reprogramming observed in *mpk4* included the specific upregulation of genes related to stress responses, cell death and SA production (Additional file 2: Table S2), in agreement with previous transcriptome data of adult plants [16]. Strikingly, approximately half of the genes that were differentially expressed in *mpk3* (51% of downregulated and 60% of upregulated genes) displayed a similar regulation in *mpk4* (Figure 1A and Additional file 3: Table S3). Within this group of 286 commonly regulated genes in *mpk3* and *mpk4*, genes controlling glucosinolate biosynthesis were upregulated and genes responding to sugar and amino acid metabolism were downregulated. In *mpk6*, 10 out of the 45 upregulated genes are chloroplast genes encoding regulators of photosynthesis and light reactions (Additional file 2: Table S2). As MAPKs are known to regulate MAMP-induced transcriptional responses, we wondered if the absence of one kinase would trigger basal changes in the transcriptome that resemble those triggered by flg22 treatment. Only a small subset of the differentially regulated genes in *mpk3*, *mpk4* and *mpk6* in mock-treated conditions was similarly regulated in Col-0 after a 30 min treatment with flg22 (Additional file 4: Figure S1). As expected, *mpk4* showed the highest overlap with the flg22-induced response but this represented only 24% of the differentially expressed genes (292 of 1,235 genes). This indicates that the basal transcriptome changes observed in these mutants do not mimic the transcriptional reprogramming triggered in Col-0 in response to flg22.

**Table 1:** Number of differentially expressed genes in the different transcriptome comparisons of the microarray analysis

**Comparison**	**Genes up**	**Genes down**
*mpk3* vs Col	305	191
*mpk4* vs Col	969	265
*mpk6* vs Col	45	16
*mpk3* + flg22 vs *mpk3*	1,519	877
*mpk4* + flg22 vs *mpk4*	1,442	634
*mpk6* + flg22 vs *mpk6*	1,468	690
Col + flg22 vs Col	1,529	862

#### Identification of MAPK-dependent genes in the flg22-triggered transcriptional reprogramming

We next analysed the transcriptome changes in response to a 30 min treatment with flg22. Col-0 reacted to the flg22 treatment with the upregulation of 1,529 genes, enriched in GO terms involved in signalling, enzymatic functions and with membrane or cell periphery targeting, in agreement with a coordinated response to an extracellular pathogen-derived signal (Additional file 5: Table S4). The downregulated genes (962 genes) showed enrichment in genes involved in hormone metabolism and signalling, RNA metabolism, transcription and response to sugar and were targeted to different subcellular compartments (Additional file 5: Table S4). These observations are in line with previous analyses of the transcriptional responses of wild type plants to flg22 [4,40,41].

We then assessed the proportion of genes that loose totally or partially their flg22-dependent regulation in the *MAPK* mutants by assessing the number of genes with at least 1 log ratio difference in the flg22-induced expression in each *MAPK* mutant as compared with Col-0 (Table 1 and Additional file 6: Table S5). We observed that 36% of the flg22-upregulated genes and 68% of the flg22-downregulated genes were affected in at least one *MAPK* mutant (Figures 1B). This revealed that besides the known role of *MPK3*, *MPK4* and *MPK6* in regulating gene induction, the three kinases have a major role in flg22-induced gene repression. As 82% of the *MAPK*-dependent flg22-induced genes and 93% of the *MAPK*-dependent flg22-repressed genes are not affected in *mpk3*, *mpk4* or *mpk6* in control conditions (Additional file 4: Figure S1) the reduction in the flg22 response cannot be explained by the basal transcriptome changes observed in the mutants in the absence of stress.

#### MPK4 positively regulates flg22-upregulated genes

We next assessed the contribution of each *MAPK* to the observed flg22-induced gene upregulation (Additional file 5: Table S4). Within the *MAPK*-dependent flg22-upregulated genes, the majority of genes (63%) showed differential regulation in only one kinase mutant and strikingly, we found that 52% (281/544 genes) are differentially regulated only in *mpk4* (Figure 1C, Additional file 6: Table S5). Only 24% of these *MPK4*-regulated genes can be attributed to a basal upregulation in untreated *mpk4* (Additional file 4: Figure S1A and Additional file 7: Figure S2). This subset of upregulated genes in untreated *mpk4* was enriched in GO terms associated with immune responses, cell death, SA, JA and ROS (Additional file 8: Figure S3D). Interestingly, many of the genes showing reduced flg22 induction in *mpk4* and not affected in mock-treated samples were associated to ET biosynthesis and signalling (Additional file 8: Figure S3B), which points to a positive role of *MPK4* in mediating the flg22-induced transcriptional reprogramming of the ET pathway. This observation fits with previous data suggesting a role for *MPK4* in mediating the induction of the JA- and ET-responsive gene *PDF1.2* in response to *P. syringae* effectors or hormone treatments [16,17,42]. The group of flg22-induced *MPK4*-dependent genes encode important regulators of plant defence such as the cell death inhibitor BAP1 [43], the calcium-dependent protein kinase CPK5 [44], the exocyst complex subunit EXO70B2 [45], the cyclic nucleotide-gated ion channel CNGC11 [46] or the BAK1-like receptor kinase BKK1/SERK4 [47].

#### MPK3, MPK4 and MPK6 equally contribute to regulate flg22-downregulated genes

We next analysed the participation of each *MAPK* in the regulation of the *MAPK*-dependent flg22-repressed genes. We found that 47% (274/586 genes) of the flg22-repressed genes lose partially or completely their regulation in *mpk3*, *mpk4* and *mpk6* (Figure 1D and Additional file 6: Table S5). Among these flg22-repressed *MAPK*-dependent genes we found enrichment in GO terms related to sugar response and the metabolism of the branched-chain amino acids (BCAA) leucine, isoleucine and valine. This group of genes is highly co-regulated and, in some cases, constitutively repressed in *mpk3* and *mpk4* (Additional file 3: Table S3 and Additional file 6: Table S5). Interestingly, isoleucine and other BCAA-related metabolic products are involved in the homeostasis of defence hormones, as for example isoleucic acid (ILA) that induces the SA pathway and resistance against *P. syringae*[48]. This highlights a role for the three *MAPKs* in repressing primary metabolic pathways in response to flg22 that may impact hormone metabolism. In addition, *mpk4* showed specific deregulation of a subset of genes involved in rRNA metabolism and in regulating transcriptional responses during morphogenesis, hormone responses and circadian rhythm, (Additional file 6: Table S5).

### Functional analysis of transcriptome data through clustering of co-regulated genes

The differential analysis performed on the transcriptome data identified genes with statistically significant differential expression but did not reveal whether the genes are regulated by common regulators. To identify genes that behave similarly across the seven comparisons, we performed a co-expression analysis based on model-based clustering. Hereby, genes with similar expression patterns are grouped in clusters that may share a similar regulatory protein or mechanism. In contrast to a clustering method based on a metric distance, like K-means or hierarchical clustering, model-based clustering assumes that the data are generated by a finite mixture of distributions. Hence, the clustering is done with a global point of view and provides a statistically rigorous framework to determine the cluster numbers and the gene assignments [49]. For this analysis, we considered probes that were differentially expressed in at least one of the seven comparisons according to the Bonferroni *P* value adjustment to limit the number of false positives. This corresponded to a total of 4,378 probes representing 4,177 genes. The clustering method found 29 clusters of co-expression and 1,928 probes corresponding to 1,876 genes were assigned into the clusters after a classification based on a threshold Maximum A Posteriori rule (Additional file 9: Figure S4 and Additional file 10: Table S6). For the biological interpretation of the analysis, for each cluster and for each comparison, the percentage of genes differentially expressed according to Bonferroni is indicated on the top of the cluster profiles. We considered for further analysis those clusters where more than 50% of the genes were differentially expressed in at least one comparison. For this reason, clusters 1 and 17 were excluded from the interpretation.

**Figure 2:**
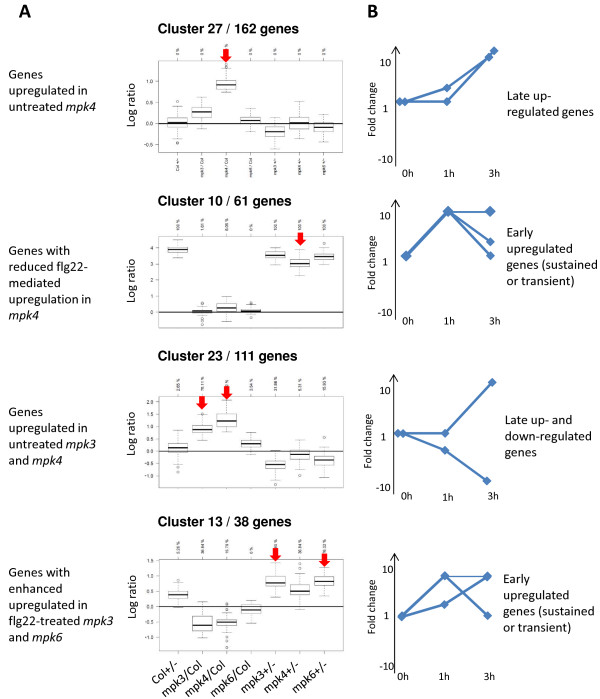
**Selection of clusters displaying interesting gene co-regulation. (A)** Profiles of the indicated clusters displaying the fold change expressions in the seven comparisons. The sentences on the left and the red arrows indicate the main message of the cluster. Y scale is log ratio. On the X scale, the following comparisons are shown: Col-0 + flg22 *vs.* Col-0, *mpk3 vs.* Col-0, *mpk4 vs.* Col-0, *mpk6 vs.* Col-0, *mpk3* + flg22 *vs. mpk*3, *mpk4* + flg22 *vs. mpk4*, *mpk6* + flg22 *vs. mpk6*. Profiles are represented as boxplots, where the bottom and top of the box are the first and third quartiles and the band inside the box is the median. Data not included between the whiskers are represented by a dot. On top is indicated the percentage of genes differentially regulated (*P* value <0.05) in the different comparisons. ‘-’ indicates mock treatment and ‘+’ indicates flg22 treatment. **(B)** Kinetic behaviour of the cluster as predicted by the comparison with ‘late’ flg22-regulated genes.

**Figure 3:**
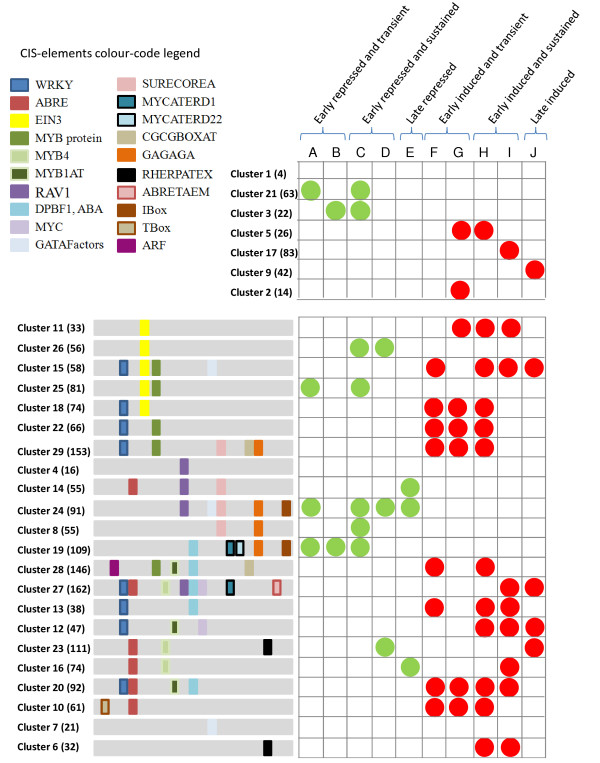
**Representation of the cis-element enrichment and the expression kinetics in the different clusters.** The left panel shows clusters sharing cis-elements. The cluster number and the number of genes of each cluster (in brackets) are shown on the left. The right panel represents the kinetic behaviour of the clusters as predicted by the comparison with publicly available data on ‘late’ flg22-regulated genes. Class F genes are induced after 1 h flg22 treatment and return to basal level after 3 h. Class G genes are induced after 1 h flg22 treatment and induced to a lesser extent at 3 h after treatment. Class H genes are induced after 1 h flg22 treatment and to a same extent at 3 h. Class I genes are induced after 1 h flg22 treatment and show further induction at 3 h. Class J genes are not induced at 1 h but show upregulation 3 h after flg22 treatment. Class A, B, C, D, E show the same tendencies but with flg22-induced downregulation. No cis-element enrichment was found for clusters 1, 2, 3, 5, 9, 17, 21. Clusters 1, 4 and 7 do not show significant enrichment in ‘late’ flg22-regulated genes. For detailed explanation, see Additional file 11: Figure S5.

We obtained three major groups of clusters: (1) clusters with genes modulated by flg22 in Col-0 (15 clusters); (2) clusters with genes affected only in *mpk4* under standard growth conditions (6 clusters); and (3) clusters showing differential expression in the *MPK3*, *MPK4* or *MPK6* mutants but not in Col-0 (6 clusters). Four representative clusters with interesting profiles are shown in Figure 2. Among the 15 clusters with flg22-regulated genes in Col-0, 10 group flg22-induced genes (Clusters 2, 5, 10, 11, 15, 18, 20, 22, 28, 29) and five group flg22-downregulated genes (Clusters 3, 19, 21, 25, 26). We then found five (Clusters 4, 6, 7, 12, 27) and one (Cluster 9) clusters that are defined by genes up- and downregulated, respectively, only in *mpk4* in standard growth conditions. We wondered whether those differentially regulated genes in *mpk4* could be regulated by flg22 at other time points not analysed in our study. To assess this, we made use of a microarray analysis performed in similar conditions as ours, which identified genes differentially expressed in Col-0 seedlings at 1 and 3 h after treatment with 1 μM flg22 [40]. Hereafter, we refer to these genes as ‘late’ flg22-regulated genes, which we grouped into different classes according to their expression kinetics (Figure 2 and Additional file 11: Figure S5). Out of the five clusters showing upregulation only in *mpk4*, three clusters (Clusters 6, 12, 27) showed enrichment in late flg22-induced genes (Figure 2 and Additional file 12: Figure S6). The genes of these three clusters and those of cluster 11 (flg22-upregulated genes induced in untreated *mpk4*) were also induced at 1 and 3 h, suggesting that the genes induced in untreated *mpk4* correspond to late flg22-upregulated genes. In contrast, clusters 10, 18, 20 and 29 group flg22-upregulated genes in Col-0 that loose part of their flg22-regulation in *mpk4* and are not differentially regulated in untreated *mpk4* (Additional file 13: Figure S7). Interestingly, these clusters were strongly enriched for early induced genes (Figures 2 and 3).

**Figure 4:**
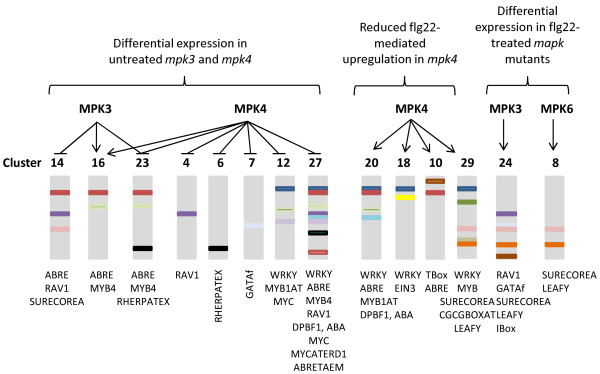
**Cooperative and Specific and roles of MPK3, MPK4 and MPK6 as revealed by cluster analysis.** Under standard growth conditions or in response to flg22, different MAPKs appear to play specific roles in the control of distinct clusters. This regulation may be under the control of the indicated enriched CIS-elements.

Clusters 8, 13, 14, 16, 23 and 24 showed a differential regulation in the MAPK mutants with or without flg22 treatment while they were not regulated by flg22 in Col-0. Specifically, the clustering approach revealed one cluster with genes upregulated in untreated *mpk3* (Cluster 14) and, as observed in the differential analysis, two clusters with similar regulation in untreated *mpk3* and *mpk4*: cluster 23 displays upregulated genes and cluster 16 downregulated genes. Therefore, the clustering approach confirmed the transcriptional similarities between unchallenged *mpk3* and *mpk4* already observed in the differential analysis. Cluster 23 grouped 86 of the 183 genes commonly upregulated in *mpk3* and *mpk4* and cluster 16 grouped 42 of the 103 genes commonly downregulated in the two *MAPK* mutants (Additional file 3: Table S3). Network building of known co-expressed genes in the cluster 23 revealed genes involved in flavonol metabolism and genes coding for enzymes that control glucosinolate production (Additional file 14: Figure S8 and Additional file 10: Table S6). The genes controlling BCAA metabolism were found within cluster 16 of downregulated genes in *mpk3* and *mpk4* (Additional file 15: Figure S9 and Additional file 10: Table S6). Interestingly, three clusters contained genes that are not affected in flg22-treated Col-0 samples but are differentially expressed in one of the *MAPK* mutants after flg22 treatment. Cluster 8 shows specific downregulation in flg22-treated *mpk6*, cluster 24 downregulation in flg22-treated *mpk3* (and to a lesser extent *mpk4* and *mpk6*) and cluster 13 flg22-triggered upregulation in both *mpk3* and *mpk6* (and *mpk4* to a lesser extent). Therefore, these clusters contain genes whose expression is maintained unchanged upon flg22 treatment due to the concerted action of these three kinases. Interestingly, the genes contained in these clusters are similarly regulated in Col-0 at the later time points of 1 or 3 h after flg22 treatment (Figure 4 and Additional file 16: Figure S10). This suggests that the MAPKs are not only required to regulate the rapid transcriptional responses to flg22 treatment, but also to prevent premature regulation of flg22-responsive genes.

#### Analysis of cis-elements

The clustering approach allows the identification of genes showing similar expression patterns, thus putatively controlled by the same upstream regulators. Therefore, we used the promoters of the genes in each cluster to assess the enrichment of known cis-elements [50]. Additional file 17: Table S7 compiles all data concerning the cis-element enrichment analysis, the size of the clusters and the number of genes that presents each given cis-element. A schematic representation of this analysis is presented in Figures 3 and 4. Many clusters containing flg22-induced genes contain promoters with enrichment in W-boxes bound by WRKY transcription factors. Several WRKY transcription factors are known MAPK substrates and play important roles in stress-related transcriptional reprogramming in plants [51]. Our comparative analysis with the kinetic data from Denoux et al. [40] also indicates that six out of the eight clusters enriched for early and transiently flg22-activated genes contain W-boxes, suggesting that these transcription factors function in the early phases of flg22-mediated gene regulation. MYB binding sites were mostly present in clusters containing flg22-induced genes and were associated in three out of five clusters with WRKY binding sites (Clusters 15, 22 and 29). Until now, MYB51 is the only transcription factor of this family that has been reported to have a role in MAMP-triggered immunity [52], while other MYB factors are involved in different plant defence mechanisms [53]. Interestingly, protein microarrays identified several WRKY and MYB factors as putative targets of MPK3, MPK4 and MPK6 [36]. We found binding sites of the ET-related transcription factor EIN3 enriched in five clusters containing genes either up- or downregulated by flg22. This factor is regulated through direct phosphorylation by MPK3 and MPK6 and plays an important role in the transcriptional control of immune signalling components such as *SID2* and *FLS2*[54-56]. The classical ABA-responsive ABRE motifs were poorly associated with flg22 transcriptional regulation, but we found ABRE-related binding sites called DPBF1-binding elements that were associated with many clusters responding to flg22. DPBF1 belongs to the A-group of bZIP transcription factors, which are mostly related to ABA signalling [57], and DPBF2 was found in protein microarrays as putative MPK4 and MPK6 substrates [36]. Among the clusters displaying genes differentially regulated in *mpk3* and *mpk4*, we found enrichment in a motif bound by RAV1, an AP2/EREBP transcription factor. Interestingly, these clusters are not regulated by flg22 in Col-0 and they group genes that are upregulated in untreated *mpk3* (Cluster 14) or *mpk4* (Clusters 4 and 27) or that present an altered expression pattern in flg22-treated *mpk3* (Cluster 24). The activity of this transcription factor is induced by mechanical stimuli [58] and may therefore be negatively controlled by *MPK3* and *MPK4*. Overall, the clustering approach coupled with the cis-element analysis allowed us to predict the function of specific transcription factors in the regulation of *MAPK-*dependent genes upon flg22 treatment.

### Construction of gene interaction networks and identification of putative regulators of the MAPK-dependent transcriptional reprogramming

We then capitalised on the transcriptome data to build a gene network that is based on publicly available experimental data and displaying validated transcription factor-target and protein-protein interactions (see Material and Methods) (Additional file 18: Figure S11). This approach allows the identification of regulators which are not transcriptionally regulated and are therefore not identified through conventional transcriptome analysis. The absence of transcriptional regulation in response to flg22 is not a detrimental criterion for biological relevance, as rapid transcriptional reprogramming responses are usually controlled by preformed factors that are post-translationally regulated. Among the different hubs we found known regulators of immune responses such as the calmodulin-like protein CML9 [59] (Additional file 18: Figure S11B) and the calcium-dependent kinase CPK11 [60] (Additional file 18: Figure S11C). CML9 belongs to cluster 29 of flg22-upregulated genes and previous protein microarray experiments showed that it interacts with three other proteins of this cluster: two leucine-rich repeat protein kinases (AT3G02880 and AT3G28450) and the cytoplasmic kinase CAST-AWAY/KIN4 (AT4G35600) [61,62]. The protein microarray also showed that these three CML9-interacting proteins share seven common interacting proteins, which are all calmodulin-like proteins [61]. Interestingly, the kinase CAST-AWAY/KIN4 is a putative MPK6 phosphorylation substrate [36], suggesting the existence of a highly interconnected network related to Ca^2+^ signalling that may be regulated by MAPKs during immune responses. The transcription factors HY5, PIF1 and AP2 involved in different aspects of plant development were also revealed as hubs (Additional file 18: Figure S11). Indeed, HY5 and PIF1 are important regulators of the transcriptional reprogramming that occurs during light-regulated processes, which are primarily regulated post-transcriptionally in response to light [63,64]. Analysis of the genes that are connected to HY5 and may constitute transcriptional targets, positioned HY5 upstream of the clusters 10 and 20 of early flg22-regulated genes modulated by *MPK4*. A similar analysis placed PIF1 upstream of cluster 29 together with several other transcription factors.

### Compensatory mechanisms at the level of MAPK protein activity occur in *mpk3* and *mpk6*

**Figure 5:**
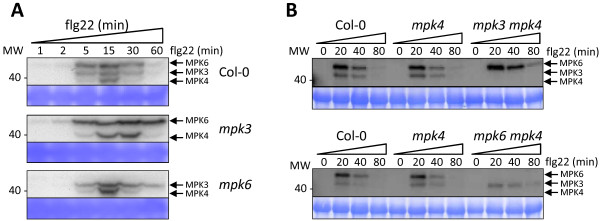
**Flg22-induced activation of MPK3, MPK4 and MPK6 in Col-0, *mpk3*, *mpk4 *and *mpk6 *single and in *mpk3 mpk4 *and *mpk6 mpk4 *double mutant plants.** Western blot analysis of Col-0, *mpk3* and *mpk6* plants **(A)** and Col-0, *mpk4*, *mpk3 mpk4* and *mpk6 mpk4* plants **(B)** at the indicated time-points after flg22 treatment, using an anti-pTpY antibody to detect activated MPK3, MPK4 and MPK6. The arrows indicate the activated forms of MPK3, MPK4 and MPK6. Blots were stained with Coomassie blue and the protein band corresponding to the RuBisCO large subunit shows equal loading.

The complex relationships between the *MAPKs* revealed by the transcriptome analysis suggested that the absence of one MAPK could influence the function of the other MAPKs. We therefore analysed the flg22-induced MAPK activities in Col-0, *mpk3*, *mpk4* and *mpk6* using an anti-pTpY antibody that recognises the dual phosphorylated activation loop of MAPKs (TEY motif). Interestingly, *mpk3* showed higher and longer activation of MPK4 and MPK6 in response to flg22 treatment, whereas *mpk6* displayed higher and longer activation of MPK3 and MPK4 (Figure 5A). In contrast, despite the increased flg22-induced MPK3 and MPK6 activities observed in mutant plants of the upstream kinase MEKK1 [15], the absence of *mpk4* did not have an impact on the flg22-induced MPK3 or MPK6 activities (Figure 5B). To further assess the impact of *mpk4*, we generated and analysed *mpk3 mpk4* and *mpk6 mpk4* double mutants. Interestingly, while the double mutant plants resembled *mpk4* phenotypically (Additional file 19: Figure S12), they showed flg22-induced MAPK activities that resembled the respective *mpk3* and *mpk6* single mutants (Figure 5B). We concluded that MPK4 does not influence the regulation of MPK3 and MPK6 activities. The *mpk3 mpk6* double mutant was not included in this analysis due to its embryo lethal phenotype [22]. In all experiments, mock-treated samples showed no signal or only weak MPK6 activity (data not shown). Importantly, the observed differential regulation of the kinase activities were not due to different MAPK protein levels (Additional file 20: Figure S13). These results indicate that MPK3 regulates MPK4 and MPK6 activities whereas MPK6 regulates MPK3 and MPK4 activities. The observed regulation could be accomplished through a direct MAPK-MAPK phosphorylation event that could negatively regulate the MAPK activities and therefore explain an enhanced kinase activity in the mutant backgrounds. To assess this hypothesis, we tested if wild type and constitutively active versions of MPK3, MPK4 and MPK6 [38] could directly phosphorylate kinase-dead versions of MPK3 or MPK6. Whereas all wild type and constitutively active MAPK proteins showed kinase activity on the MBP substrate (Additional file 21: Figure S14A) and displayed autophosphorylation (Additional file 21: Figure S14B), none of the MAPKs phosphorylated the dead versions of MPK3 or MPK6 in *in vitro* kinase assays (Additional file 21: Figure S14B). We concluded that MPK3 and MPK6 regulate MAPK activities through an indirect mechanism that may involve upstream kinases or phosphatases. As we did not detect any phosphorylation of the dead MPK6 or dead MPK3 by the respective wild type or constitutively active kinase versions, these data show that MAPK autophosphorylation is caused by intramolecular and not intermolecular phosphorylation.

### Flg22-induced SA production is enhanced in *mpk3*

**Figure 6:**
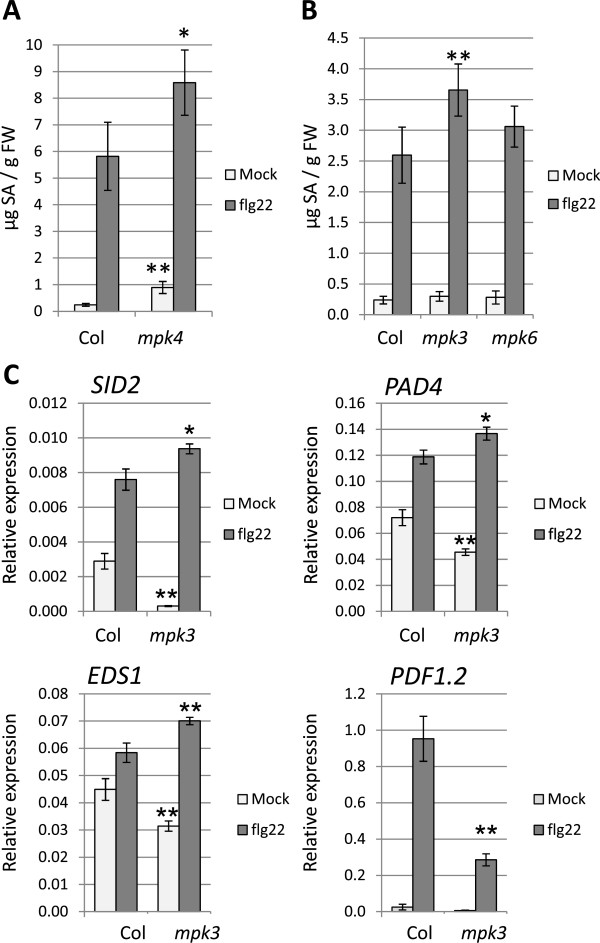
***mpk3 *shows enhanced flg22-mediated activation of SA-mediated defences.** SA accumulation 24 h after mock-treatment or treatment with 1 μM flg22 in Col-0 and *mpk4***(A)** and in Col-0, *mpk3* and *mpk6* seedlings **(B)**. Bars are means ± SD. **(C)** qPCR analysis of the expression of SA marker genes *SID2*, *EDS1* and *PAD4* and the ET/JA marker gene *PDF1.2* in Col-0 and *mpk3* seedlings 24 h after treatment with 1 μM flg22. Transcript accumulation is expressed relative to the reference gene *ACTIN2*. Bars represent means ± SE of three independent biological replicates and each replicate is composed of three technical replicates. Stars indicate significant difference with Col-0 under the same conditions based on a two-tailed Student’s ttest. **P value <0.01 and *P <0.05.

*mpk4* displays constitutive activation of the SA pathway and enhanced resistance to biotrophic pathogens [16]. Since *mpk3* and *mpk4* transcriptomes presented a significant overlap in unchallenged conditions, we tested whether the SA pathway was also activated in *mpk3*. Contrary to *mpk4*, we observed no differential expression in genes involved in SA biosynthesis and signalling in either control or flg22-treated *mpk3* plants (that is, *SID2*, *EDS1*, *PAD4*, *ACD6*, *NDR1*, *ALD1*, *EDS5* and *NPR1*[65]). However, flg22-mediated induction of *SID2* occurs at later time points and is not the rate limiting step in flg22-induced SA accumulation [40,66]. To assess whether *MPK3* could play a role in the regulation of the SA pathway, we quantified SA accumulation in resting and flg22-challenged seedlings in a similar way as for the expression analyses. Flg22 leaf infiltration induces a 10-fold increase in SA levels in adult Arabidopsis plants [66], but the flg22-induced SA accumulation in Arabidopsis seedlings has not been reported yet. In response to 1 μM flg22 treatment, Arabidopsis wild type seedlings displayed a 10- to 20-fold increase in total SA (Figure 6A and 6B). Whereas *mpk4* seedlings displayed enhanced SA accumulation in resting conditions and higher flg22-induced SA levels compared to Col-0 (Figure 6A), *mpk3* showed wild type levels of SA in resting conditions but enhanced flg22-induced SA levels 24 h after treatment (Figure 6B). The increased SA accumulation correlated with the moderately increased flg22-induced transcript levels of the SA biosynthetic gene *SID2* and the SA signalling genes *EDS1* and *PAD4* (Figure 6C). On the contrary, the JA and ET marker gene *PDF1.2* displayed reduced flg22-induced accumulation in *mpk3*. These results suggest that the observed similarities in the transcriptomes of unchallenged *mpk3* and *mpk4* are not due to a similar deregulation of the SA pathway. We concluded that in contrast to the role of MPK4 that regulates constitutive and inducible SA levels, MPK3 has a role in dampening the SA pathway upon perception of MAMPs and possibly pathogens.

### Flg22-induced callose deposition is reduced in *mpk3*

**Figure 7:**
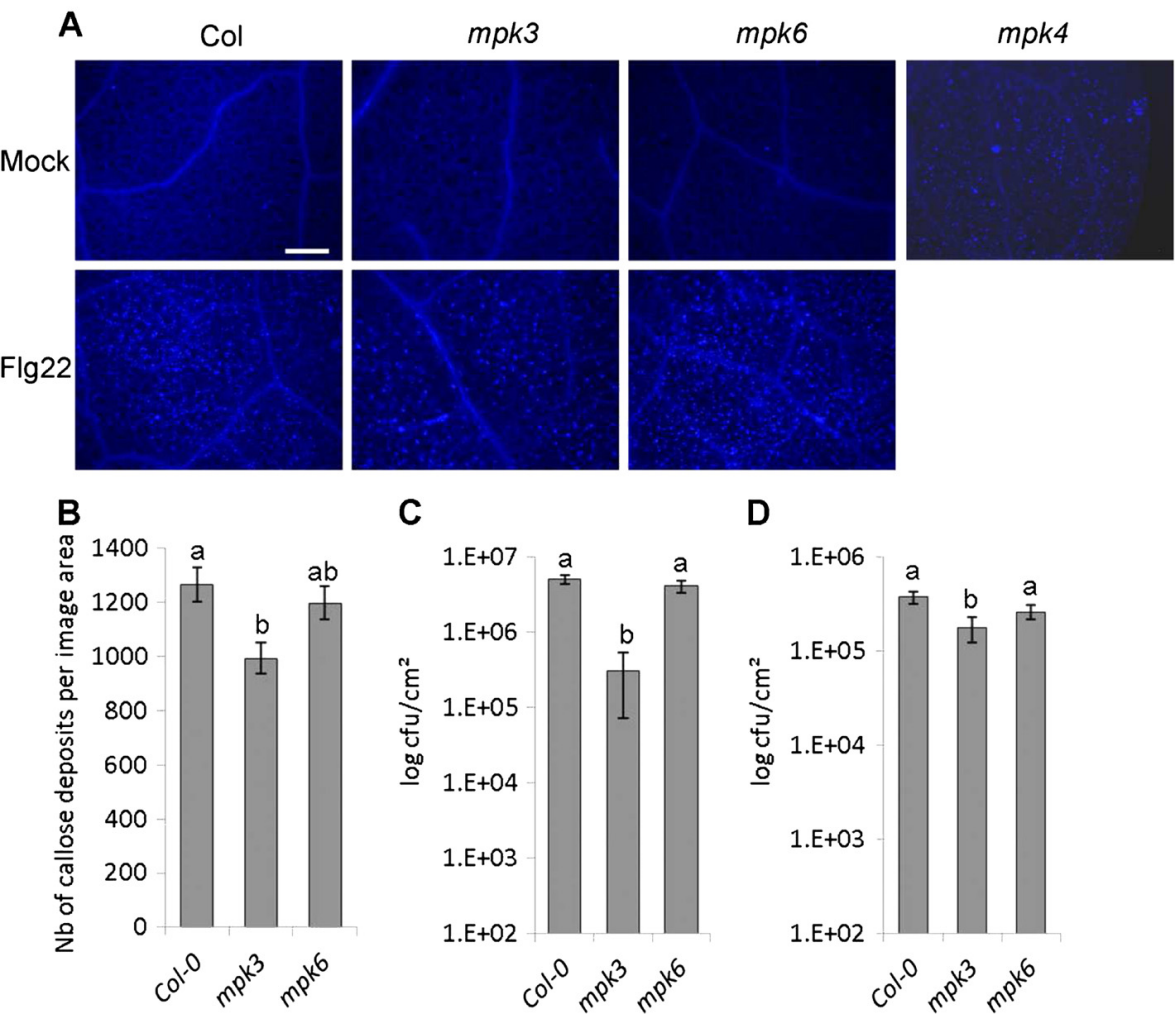
***mpk3 *adult plants show reduced flg22-induced callose accumulation and enhanced resistance to virulent *P. syringae. *(A)** Pictures of aniline-blue stained leaves of the indicated phenotypes. Leaves were either untreated (*mpk4*), infiltrated with mock (H_2_0) or a 1 μM flg22 solution for 24 h. Bar is 200 μm. **(B)** Relative quantification of callose deposition in leaves of Col-0, *mpk3* and *mpk6* after infiltration of 100 nM flg22. **(C)***Pst* DC3000 bacterial titres 3 days post spray-inoculation. Bars are means ± SD (n = 5). **(D)***Pst* DC3000 bacterial titres 3 days post inoculation by syringe-infiltration. Bars are means ± SD (n = 4). Letters indicate significant difference based on a Kruskal & Wallis test (α <0.05).

We next analysed the *MAPK* mutants for callose deposition, another defence hallmark induced by flg22 and proposed to be regulated by MAPK cascades. Previous studies on transgenic plants expressing the *P. syringae* effector HopAI1 or MKK5^DD^, which, respectively, inactivate and activate both MPK3 and MPK6, suggested that these two MAPKs are necessary for callose accumulation [21]. In addition, *mekk1* plants show constitutive callose deposition [15]. In agreement with this, we found that *mpk4* plants also display constitutive callose accumulation (Figure 7A). The transcriptome analysis showed that *mpk3* and *mpk4* share the upregulation of certain genes involved in the indole glucosinolate-dependent production of callose (Additional file 14: Figure S8). We therefore tested if MPK3 could be involved in the regulation of this inducible defence mechanism. In contrast to *mpk4*, *mpk3* and *mpk6* displayed no constitutive callose accumulation (Figure 7A), but infiltration of flg22 into leaves of adult plants led to a reduced number of callose deposits in *mpk3* whereas *mpk6* had an intermediate behaviour between wild type and *mpk3* (Figure 7B).

### *mpk3* displays reduced susceptibility to virulent *Pseudomonas syringae*

To assess the impact of the altered flg22-induced transcriptional reprogramming, SA production and callose deposition in *mpk3* with respect to disease resistance, we challenged *mpk3* and *mpk6* plants with the virulent bacteria *P. syringae* pv. *tomato* DC3000 (*Pst* DC3000). The analyses were performed on adult plants and therefore *mpk4* mutant plants were not included due to their dwarf phenotype. In spray inoculated plants, *mpk3* showed significantly lower bacterial titres while *mpk6* behaved like Col-0 (Figure 7C). Given that both *mpk3* and *mpk6* are impaired in flg22-induced stomatal closure [32], we assessed whether the enhanced resistance in *mpk3* was related to post-invasive resistance by quantifying bacterial growth after inoculation via syringe infiltration that surpasses the stomatal barrier. Using this infection method, *mpk3* plants showed weak but significant reduced susceptibility to *P. syringae* as compared to Col-0 and *mpk6* (Figure 7D), indicating that MPK3 also plays a role in modulating post-invasive disease resistance in mesophyll cells.

## Discussion

We performed a comprehensive analysis of early and late responses triggered by flg22 in *mpk3*, *mpk4* and *mpk6*, which revealed new roles for these immune-related MAPKs in stress signalling but also in unchallenged tissues. In untreated conditions, *mpk6* displayed minor transcriptional changes, while we unexpectedly found that *mpk3* and *mpk4* shared the differential regulation of an important set of genes. Several of the genes differentially regulated principally in *mpk4* but also in *mpk3*, were identified as ‘late’ flg22-regulated genes in previous reports [40]. This suggests that MPK4 and MPK3 function together in unstressed conditions to prevent misregulation of defence genes and to inhibit a premature reprogramming of flg22-regulated genes. The fact that MPK4 shares 56% phosphorylation targets with MPK3 and 28% with MPK6 [36], further supports this hypothesis. Nevertheless, even if *mpk3* and *mpk4* share common differentially regulated genes under normal growth conditions, *mpk3* does not show the developmental defects observed in *mpk4* at the adult stage. Therefore, these data reveal similarities but also fundamental differences in the roles of MPK3 and MPK4. In contrast to the role of MPK4 in repressing basal and pathogen-induced SA and ROS accumulation [16,38], *MPK3* seems to dampen SA and ROS production only after pathogen challenge. The cause of this difference may rely on the nature of the genes constitutively upregulated in *mpk4* (related to SA and cell death) and the MPK4-mediated negative regulation of the MAPKKK MEKK2 and the NB-LRR SUMM2 [11-13]. In response to flg22 treatment, we observed that one-third of early flg22-regulated genes are differentially regulated in at least one of the three *MAPK* mutants. Among these genes, two-thirds are downregulated and are equally controlled by *MPK3*, *MPK4* and *MPK6* suggesting a cooperative activity of the three kinases in gene repression. With respect to the flg22-induced genes, we unexpectedly found an important proportion of flg22-induced genes showing compromised regulation in *mpk4* in response to flg22, which were not differentially regulated in untreated *mpk4*. This reveals that *MPK4*, usually considered as a negative regulator of defence responses, is also a master regulator of early flg22-induced transcriptional activation. In summary, these observations indicate the existence of MAPK specific and cooperative functions in gene regulation. In agreement with this concept, there are transcription factors known to be regulated by one (that is, ERF104 [34]), two (that is, ERF6 [26]) or the three MAPKs (that is, WRKY33 [67,68]).

The clustering and cis-element analyses and the construction of interaction networks, allowed us to identify putative regulators of the MAPK-dependent transcriptional responses. The clustering and cis-element analyses revealed several WRKY and MYB transcription factors as putative downstream factors controlling MAPK-dependent transcriptional reprogramming. This is in agreement with WRKY and MYB factors being known MAPK targets involved in stress responses [36,67,68]. Interestingly, the clustering analysis also revealed EIN3 binding sites in clusters of flg22-upregulated or downregulated genes. EIN3 is involved in the induction and repression of important immune components such as *FLS2* and *SID2*, respectively [55,56]. As EIN3 is a phosphorylation target of MPK3 and MPK6 involved in the regulation of the flg22-induced transcriptional reprogramming [54,56], it seems possible that EIN3 mediates the *MPK3*-dependent *SID2* repression as well as other *MAPK*-dependent transcriptional changes. As a complementary approach, we used the interaction network analysis that reveals putative regulatory hubs that are not transcriptionally regulated. Rapid transcriptional reprogramming responses are usually controlled by preformed transcription factors that are post-translationally regulated. Such key transcriptional regulators are expected to be present in the FLS2-mediated signalling pathway but are still unknown. In our analysis, we found two light-regulated transcription factors, HY5 and PIF1 as putative preformed transcription factors that may be involved in the FLS2 pathway. Indeed, HY5 is an important regulator of photomorphogenesis that is primarily regulated post-transcriptionally by protein degradation in response to light [63] and PIF1 is regulated by phosphorylation and other post-translational modifications in response to blue light [64]. Interestingly, a recent report showed that HY5 and PIF1 interact in Arabidopsis nuclei and coordinately regulate ROS and stress-related genes in response to light [69], suggesting that HY5 and PIF1 could modulate MAPK-dependent gene regulation of stress-related genes.

The transcriptome analysis revealed cooperative roles for the three MAPKs and prompted us to analyse whether the absence of one MAPK could influence the functioning of the other two MAPKs. Our biochemical analysis indeed revealed that MPK3 and MPK6 influence the activities of the other two stress-related MAPKs. In *mpk3,* there is longer and stronger activation of MPK4 and MPK6, whereby in *mpk6* there is longer and stronger activation of MPK3 and MPK4. In contrast, *mpk4* did not show differential MAPK regulation. Despite the enhanced MPK3 and MPK6 activities observed in a mutant of the upstream kinase MEKK1 [15], no differential regulation of flg22-induced MPK3 and MPK6 was detected in the double mutant of the downstream MKK1 and MKK2 [9]. These data indicate that the differential regulation of MPK3 and MPK6 observed in *mekk1* is independent of the downstream kinases. This unexpected finding sheds light on the complex cross-talk between MPK3, MPK4 and MPK6 during FLS2-mediated signalling. The intensity and duration of MAPK activities are key signatures, which can trigger different responses. Indeed, plant immune responses lead to transient MAPK activation during PTI and sustained MAPK activities during ETI [8,70]. Thus, it is possible that *mpk3* and *mpk6* phenotypes are not only due to the loss of function of one MAPK but also to the prolonged activities of the two other stress-induced MAPKs. In light of these results, it seems necessary to reconsider previous data obtained with *mpk3* and *mpk6* mutants, as certain phenotypes attributed to the loss of function of one MAPK may be due to the increased activity of other stress-induced MAPKs. The enhanced kinase activities in the respective MAPK knock out mutants may alter a number of properties of the affected MAPKs, such as their subcellular localization, substrate specificity, stability or complex formation. These changed properties may in turn compensate for the knocked out MAPK protein or lead to different responses.

MAPK activities are regulated by the concerted action of kinases and phosphatases. In our study we did not observe direct interaction in yeast (data not shown) or phosphorylation between the three MAPKs, which favours an indirect cross-talk mechanism. One such indirect mechanism may be mediated by protein phosphatases. Phosphatases are the major negative regulators of MAPKs and, indeed, the dual specificity phosphatase MAPK Phosphatase 1 (MKP1) and the Ser/Thr PP2C-type phosphatase AP2C1 are known regulators of the activation of MPK3, MPK4 and MPK6 in response to MAMPs or DAMPs (damage-associated molecular patterns) [27,28,71]. In animal cells, the MAPK Phosphatase 3 (MKP3) regulates the activity of the MAPKs p38 and ERK2 and forms a ternary complex with the two kinases that mediates cross regulation between both MAPK pathways [72]. A similar situation could explain the differential regulation of MAPK activities we observed in the three *MAPK* mutants. A plausible hypothesis would be that MPK3 and MPK6 regulate the activity of phosphatases that in turn regulate the activation of the other MAPKs. Indeed, MKP1 was shown to be a target of MPK6 [73] and MPK6 inactivation observed in AP2C1 overexpressing lines was partially suppressed by *mpk3*[27], suggesting that MPK3-mediated activation of AP2C1 is necessary for its phosphatase activity. In a recent phosphoproteome analysis, we identified two MKP1 phosphopeptides, with a pSP motif, whose abundance increases in response to 15 min flg22 treatment [74]. These sites are important for the regulation of MKP1 phosphatase activity and were shown to be phosphorylated by MPK6 and presumably also by MPK3 [73,75]. Our phosphoproteome analysis also identified the phosphopeptides corresponding to the MPK4 and MPK6 activation loops both in the dual and single phosphorylated states [74], supporting the idea that MKP1 and other phosphatases could play a role in the regulation of these MAPKs in response to flg22. Alternatively, the transcriptional regulation and protein turnover of the flg22 receptor FLS2 are also important determinants of the activation of the pathway [55,76]. Although the transcriptome analysis did not reveal important differences in *FLS2* expression, we cannot exclude that FLS2 transcript or protein accumulation could be differentially regulated in the *MAPK* mutants by transcriptional regulation through EIN3 or other factors.

We found that *mpk6* shows minor changes in the transcriptome and no changes in SA accumulation, callose deposition and *Pst* DC3000 susceptibility, while displaying stronger and prolonged MPK3 and MPK4 activities than wild type plants. These results suggest that either MPK6 plays minor functions in FLS2-mediated signalling or that the enhanced activities of MPK3 and/or MPK4 are able to reconstitute most MPK6 functions required in these conditions. In contrast, *mpk3* mutant displayed important transcriptome changes, enhanced flg22-triggered SA accumulation, reduced callose accumulation and reduced susceptibility to *Pst* DC3000, despite presenting enhanced MPK4 and MPK6 activities. We therefore conclude that MPK4 and MPK6 lack unique features of MPK3. While we were surprised by the phenotypes observed in *mpk3*, which is usually considered as a positive regulator of PTI and disease resistance together with MPK6, we found several indications in recent reports suggesting distinct roles for the two kinases. For example, MPK3 and MPK6 play different roles in the defence response to *B. cinerea*: while MPK3 is required for basal resistance, MPK6 contributes only to elicitor-induced resistance to the fungus [25,27]. On the other hand, previous reports showed that MPK6, and not MPK3, is necessary for deregulated stress phenotypes [28,29]. Indeed, *mkp1* mutant plants show enhanced resistance to virulent *P. syringae* and enhanced MPK6 activation, and the disease resistance was suppressed in a *mkp1 mpk6* double mutant [28]. These data suggest that the enhanced activity of MPK6 may account for the enhanced stress responses observed in *mpk3.* Unfortunately, the embryo lethality of the *mpk3 mpk6* double mutant prevents the verification of this hypothesis.

Previous data on the *P. syringae* effector HopAI1, a phosphothreonine lyase that inactivates MPK3 and MPK6, suggested that these two MAPKs regulate the flg22-induced RbohD-dependent ROS production and callose accumulation [21]. These conclusions were based on the use of transgenic plants with inducible expression of MKK5^DD^ and HopAI1, which respectively activate and inactivate the two MAPKs. Therefore these approaches did not allow distinguishing between the specific contributions of each kinase. Using *MAPK* single mutants, we and other groups could show that flg22-treated *mpk3* displays prolonged ROS production and increased growth inhibition but reduced callose deposition ([33] and this study). In contrast, *mpk6* behaved like wild type or had minor phenotypes in all assays. Recently, it was shown that HopAI1 is also capable of dephosphorylating and thereby inactivating MPK4 [13]. Nevertheless, current evidence indicates that while the MEKK1-MKK1/MKK2-MPK4 pathway inhibits basal callose accumulation (probably via repression of MEKK2 and SUMM2), it does not influence flg22-induced callose deposition [13,15,38]. This suggests that the reduced flg22-induced callose accumulation observed in HopAI1 transgenic plants and the constitutive callose accumulation in MKK5^DD^ expressing plants is due to their regulation of MPK3. Callose deposition imposes a physical barrier to pathogen penetration but its real role in resistance is still unclear. Indeed, *pmr4* mutant plants, impaired in stress-induced callose deposition, results in an over-activation of SA-mediated defence responses leading to enhanced resistance [77,78]. Therefore, the existence of a feedback regulatory mechanism was proposed, where normal activation of pathogen-induced cell wall modifications stops the activation of downstream defences, and in contrast defects in the initial defence barrier lead to over-activation of the downstream SA defence pathway. A recent network modelling approach studying Arabidopsis immune signalling, revealed an inhibitory effect of SA-signalling on flg22-induced *PMR4*-dependent callose deposition [79]. These data indicate the possibility that the reduced flg22-induced callose accumulation in *mpk3* could be due to the enhanced induced SA accumulation. On the other hand, flg22-induced RBOHD-dependent ROS production was proposed to be independent of MPK3 and MPK6 [80] and flg22-induced callose deposition is mostly RBOHD-dependent [21]. It is therefore difficult to propose a model that reconciles all published data. Network analysis further revealed a negative link between SA and MPK3. Indeed, the transcriptional reprogramming induced in *mpk3* by the PTI-inducing *Pst HrcC* bacteria showed a strong correlation with the JA/ET deficient mutants *ein2*, *dde2* and *coi1* and not with mutants in SA signalling and included an increased *SID2* expression [79,81]. These data are in agreement with our observations. Taken together, our analysis identified MPK3 as a key negative regulator of defence gene expression, flg22-induced SA signalling and disease resistance to *Pseudomonas syringae*.

## Conclusions

A comprehensive molecular and phenotypic analysis was performed for flg22-triggered responses in *mpk3*, *mpk4* and *mpk6*, revealing new roles for these immune-related MAPKs in stress signalling but also in unchallenged tissues. A genome-wide transcriptome analysis of untreated and flg22-challenged *MAPK* mutants coupled with model-based clustering, plus the construction of gene interaction networks, allowed us to identify putative regulators of MAPK-dependent transcriptional reprogramming. Altogether, this work provides evidence that MPK3, MPK4 and MPK6 possess both cooperative and specific functions in plant immune regulation and that the absence of one MAPK influences the activities of the other stress-induced MAPKs. The link between the three MAPK pathways provides an integrated mechanism to optimally coordinate the immune responses of plants.

## Material and methods

### Plant material

Arabidopsis thaliana ecotype Col-0 was used in this study. The mutants were: *mpk4-2* (SALK_056245), *mpk3* (SALK_151594) and *mpk6-2* (SALK_073907). For bacterial growth curves and callose detection assays, plants were grown on soil for 4 to 5 weeks in short day conditions (8 h light, 16 h dark), with 22°C and 65% relative humidity. For gene expression analyses, protein extraction for immunoblot analyses and SA accumulation, seedlings were grown *in vitro*. Seeds were surface sterilised and stratified for 2 days at 4°C. Seedlings were then grown for 13 days in a culture chamber at 22°C with 16 h photoperiod, on MS plates (0.5 × Murashige Skoog Basal Salts (Sigma #M6899), 1% sucrose, 0.5% agar, 0.5% MES, pH 5.7). Twenty-four hours before treatment, liquid MS (same media without agar) was added to the MS plates to facilitate the transfer of seedlings to liquid MS. Seedlings were treated with deionized water (mock) or with a final concentration of 1 μM flg22, for the required times and then frozen in liquid nitrogen. In the case of *mpk4* single mutant, *mpk3 mpk4* and *mpk6 mpk4* double mutants, the *mpk4-2* mutation was segregating. These seedlings were thus first grown vertically in MS plates with 1% agar for 7 days to isolate *mpk4*^-/-^ seedlings based on their root phenotype (thickening and shortening of the primary root [82]). Selected seedlings were then transferred to liquid MS with the growth conditions previously described and treated as the other lines at 14 days old.

### RNA extraction and RT-qPCR

For flg22-induced gene regulation, seedlings were treated with 1 μM flg22 for 1 h. RNA was extracted and DNA digested using the RNeasy plant mini kit and the RNase-Free DNase Set (Qiagen). Three different biological replicates were performed and 1 μg of each RNA was pooled to synthesize cDNA using the Superscript II enzyme (Invitrogen). Two microliters of a 100x dilution of the cDNA was used for each quantitative PCR, using a 7900 HT Sequence Detection System (Applied Biosystem) and MESA Green qPCR Mastermix Plus detection system (Eurogentec). RNA/cDNA variable inputs were corrected by normalisation to the housekeeping transcript ACT2. Error bars shown represent the standard deviations obtained from three technical replicates. Oligonucleotides used in this study for RT-qPCR are: ACT2-For 5′-CGTTTCTATGATGCACTTGTGTG-3′, ACT2-Rev 5′-GGGAACAAAAGGAATAAAGAGG-3′, SID2-For 5′-AGCTGGAAGTGACCCATCTT-3′, SID2-Rev 5′- TGGTGAACTGCAAAAACAACA-3′, EDS1-For 5′-CTCAATGACCTTGGAGTGAGC-3′, EDS1-Rev 5′-TCTTCCTCTAATGCAGCTTGAA-3′, PAD4-For 5′-TGGTGACGAAGAAGGAGGTT-3′, PAD4-Rev 5′-TCCATTGCGTCACTCTCATC-3′, PDF1.2-For 5′-GGACATGGTCAGGGGTTTGCGG-3′ and PDF1.2-Rev 5′-TGTGTGCTGGGAAGACATAGTTGC-3′.

### Transcriptome studies

Microarray analysis was carried out at the Unité de Recherche en Génomique Végétale (Evry, France), using the CATMAv6.2 array based on Roche-NimbleGen technology. CATMAv6.2 microarray slides contain 12 chambers, each containing 219,684 primers representing all the *Arabidopsis thaliana* genes: 37,309 probes corresponding to TAIRv8 annotation (including 476 probes of mitochondrial and chloroplast genes) and 1,796 probes corresponding to EUGENE software predictions. The slides also include 5,328 probes corresponding to repeat elements, 1,322 probes for miRNA/MIR, 329 probes for other RNAs (rRNA,tRNA, snRNA, soRNA) and several controls. In each chamber, probes are present in triplicates and in both strands. Three independent biological replicates of the microarray analysis were produced. For each biological repetition and each point, 14-day-old seedlings grown in long day conditions were collected and RNA samples were obtained by pooling more than 50 plants. Total RNA was extracted using Qiagen RNAeasy according to the supplier’s instructions. For each comparison, one technical replicate with fluorochrome reversal was performed for each biological replicate (that is, six hybridisations per comparison). The labelling of cRNAs with Cy3-dUTP or Cy5-dUTP (Perkin-Elmer-NEN Life Science Products) and the hybridisation to the slides were performed as previously described [83]. Two micron scanning was performed with InnoScan900 scanner (Innopsys^R^, Carbonne, FRANCE) and raw data were extracted using Mapix^R^ software (Innopsys^R^, Carbonne, FRANCE).

### Differential analysis of microarray data

For each array, the raw data comprised the logarithm of median feature pixel intensity at wavelengths 635 nm (red) and 532 nm (green). For each array, a global intensity-dependent normalisation using the loess procedure [84] was performed to correct the dye bias. The differential analysis is based on the log-ratios averaging over the duplicate probes and over the technical replicates. Hence the numbers of available data for each gene equals the number of biological replicates and are used to calculate the moderated *t*-test [85]. Under the null hypothesis, no evidence that the specific variances vary between probes is highlighted by Limma and consequently the moderated t-statistic is assumed to follow a standard normal distribution. To control the false discovery rate, we calculated adjusted *P* values using the optimised FDR approach [86]. We considered as being differentially expressed the probes with an adjusted *P* value ≤0.05. Analysis was done with the R software. The function SqueezeVar of the library limma has been used to smooth the specific variances by computing empirical Bayes posterior means. The library kerfdr has been used to calculate the adjusted *P* values. The overlap between different sets of genes was generated by the Venn diagram generator Venny [87]. The analysis to find over-represented categories in the gene sets was obtained with AmiGO [88], which is based on a hypergeometric test. Co-expression analysis was performed with ATTEDII version 6.1 using the Network Drawer tool and ‘add a few genes’ settings for co-expression and Protein Protein Interaction options [89,90]. The thickness is representative of the rank of correlation between two genes of interest via the calculation of a geometric averaged rank (MR).

### Data availability

Microarray data from this article were deposited at CATdb [91] (Project RA12-05_mut_flg_II) and GEO (Project GSE52587) according to the ‘Minimum Information About a Microarray Experiment’ standards.

### Clustering of microarray data

The dataset for the co-expression analysis was built from the results of the differential analyses. Probes with at least one Bonferroni pvalue lower than 0.05 were considered. It leads to a dataset of 4,378 probes described by seven expression differences, each one being the average of the three biological replicates. The clustering was performed with a multidimensional Gaussian mixture with unequal proportions and a component number varying from 2 to 40. Covariance matrices are constrained so that their volumes differ and their orientation and shape are equal. Estimations were done with the MIXMOD software [92] and a mixture of 29 components was selected according to the BIC criterion. Probes were assigned in the cluster for which the conditional probability is the highest and interpretation was done only for probes for which this probability is greater than 0.878. This threshold was fixed so that as many observations as possible were classified, under the constraint that the proportion of misclassified observations is controlled at a level of 5%. It is an extension of the BFDR previously described [93]. In our analysis based on 4,378 probes 1,928 probes were classified, which means that in average 96 probes were badly assigned. Cluster profiles are represented as boxplots. The bottom and top of the box are the first and third quartiles, denoted respectively Q1 and Q3. The band inside the box is the median. The ends of the whiskers represent, respectively, Q1 - 1.5 x (Q3-Q1) and Q3 + 1.5 × (Q3-Q1). Data not included between the whiskers are represented by a dot. On top is indicated the percentage of genes differentially regulated (Bonferroni *P* value <0.05) in the different comparisons.

### Detection of the cis-elements

We analysed the presence of conserved motifs in the 5′ region of genes, also known as cis-elements. The Arabidopsis promoter dataset was downloaded from FLAGdb++ based on TAIRv8 [94]. The dataset includes 27,025 promoters containing 1,000 base pairs upstream known transcription starting sites (TSSs) or upstream the ATG start otherwise. A list of 140 motifs known to be involved in stress responses was extracted from the databases PLACE [95] and AGRIS [96]. For each cluster, the presence of these motifs was identified by the Preferentially Located Motifs (PLMs) method [97]. This method determines the preferential location of each motif relative to the TSS and a functional window derived from the peak boundaries of the region in which the transcription factor binding site is over-represented. Taking into account the position of the binding site with respect to the TSS limits the rate of false positives. A motif identified by this method is a motif overrepresented at a given place regarding the TSS and is named PLM. 29 motifs were declared as PLMs among 140 motifs tested. To evaluate whether a given PLM was over-represented in a cluster with respect to the whole genome, a binomial test was performed by comparing the gene number of this cluster containing this PLM to the gene number containing this PLM in the same functional window at the genome level. PLMs with a *P* value lower than 0.01 were considered as significantly over-represented.

### GO analysis for the co-expression clusters

Gene function annotation was downloaded from TAIRv10 and the GO Slim classification for the three branches of the GO vocabulary (biological process, molecular function and cellular component) was considered. The enrichment analysis was performed by comparing the ratio of the relative occurrence of a GO term into the cluster to its relative occurrence in the genome by a hypergeometric test. A GO term was declared significantly over-represented if its *P* value was lower than 0.05.

### Gene interaction network construction

A total of 12,741 protein-protein interactions (PPI) data were extracted from: (1) Arabidopsis Interactome Consortium [98], where a matrix of 9 k × 9 k full length protein encoding ORFs were tested by yeast 2-hybrid assay and a total of 6,475 positive interactions were detected; (2) public databases: BioGRId, IntAct, TAIR and BIND (6365 experimental PPI data). Concerning the TF-target data, 769 confirmed interactions were downloaded from AtRegNet database [96]. These interaction information on protein-protein interactions (PPI) and transcription factor-target interactions were combined to the co-expression clusters using a home-made Perl program leading to a gene interaction network of 839 genes linked with 983 edges. Network visualisation and analysis were done with Cytoscape [99]. The node degree varied between 1 and 115 with a median equal to 1 and a third quartile equal to 2, meaning that the majority of the genes were few connected, and the 10 most connected genes had a degree greater than 19. For this reason we defined as regulatory hubs those proteins displaying more than 19 edges, with each edge representing a validated interaction.

### Immunoblotting

*Protein extractions*: approximately 100 mg of frozen samples were ground in liquid nitrogen using a tissue lyser (Qiagen) and metal beads. The ground material was resuspended in 200 μL of a extraction buffer containing 50 mM Tris-HCl pH 7.5, 150 mM NaCl, 0.1% NP40, 5 mM EGTA, 0.1 mM DTT (Sigma-Aldrich chemicals), protease inhibitors (Complete cocktail, Roche, and 1 mM PMSF, Sigma-Aldrich) and phosphatase inhibitors (1 mM NaF, 0.5 mM Na_3_VO_4_, 15 mM beta-glycerophosphate, 15 mM 4-nitrophenyl phosphate, Sigma-Aldrich chemicals). The suspension was centrifuged at 20,000 g for 15 min at 4°C and the supernatant was collected. Protein quantification was carried out with Bradford (Sigma-Aldrich) and BSA standard (Thermo Scientific), and the normalised protein amounts of all the samples were denatured by boiling in SDS-sample buffer at 95°C. When specified, the ground material was directly boiled at 95°C in 2× SDS-sample buffer and centrifuged at 20,000 g for 2 min. The supernatant was recovered and proteins were quantified with Amido Black 10B (Sigma-Aldrich). *Immunoblottings*: Protein samples were separated on 10% SDS-PAGE gels and transferred onto PVDF membranes (GE Healthcare). *Anti-pTpY antibody*: blots were blocked with 5% (w/v) BSA (Sigma-Aldrich) in TBST and incubated overnight at 4°C with the rabbit anti-phospho-p44/42 MAPK (Erk1/2) (Thr202/Tyr204) monoclonal antibody (Cell Signalling) at a dilution of 1/1,500. *Anti-MAPK antibodies*: Blots were blocked in 5% (w/v) non-fat dry milk in TBST and incubated overnight at 4°C with anti-MPK3 and anti-MPK4 antibodies previously described [100] at a dilution of 1/3,000, or with anti-MPK6 antibody (Sigma-Aldrich) at a dilution of 1/5,000. As secondary antibody we used the goat anti-rabbit horseradish peroxidase (HRP)-conjugated (Sigma-Aldrich) diluted to 1/20,000. HRP activity was detected with a chemiluminescent reagent (GE Healthcare) using the GeneGnome imaging system (Syngene) or clear-blue X-ray films (Thermo Scientific). Blots were stained with Coomassie blue for protein visualization. Each immunoblotting analysis shown is representative of at least two independent biological repeats.

### Purification and activity assays of recombinant MAPKs

MAPK protein expression in *E. coli*, purification, and activity assays were performed as previously described [38]. Wild type and constitutive active (Y and DE variants) variants of the MAPK proteins were His-tagged in the case of MPK4 and MPK6 and fused to peri-His-MBP in the case of MPK3 as previously described [38]. GST-tagged kinase dead variants of MPK3 and MPK6 carry mutations in the ATP binding site and were previously described [101].

### Salicylic acid quantification

Total SA was extracted as previously described [102] with the following modifications. [^14^C]SA (50 Bq, 2 GBq mmol^−1^, NEN, UK) was added to each sample to correct for losses. Samples were dried in a SC 110A Speed-Vac (Savant Instrument Inc., New York, NY, USA) and subjected to acidic hydrolysis in order to determine total SA. SA was identified and quantified by HPLC based on a comparison with the standard. SA standard was purchased from Sigma-Aldrich (Saint-Quentin Fallavier, France).

### Pseudomonas assays

Infections with *Pseudomonas syringae* pv. *tomato* (*Pst*) DC3000 were done by spray inoculation with bacterial solution at 1×10^8^ cfu/mL or by syringe-infiltration at 1×10^5^ cfu/mL. Bacterial titers were determined as previously described [103].

### Callose assays

Callose assay was performed as previously described after infiltration of leaves of adult plants with H_2_0 (mock) or 1 μM flg22 solution [40].

## Competing interests

The authors declare that they have no competing interests.

## Authors’ contributions

NFdF, AVG and HH designed research. NFdF and AVG performed callose staining, RT-qPCR and *P. syringae* infections. AVG and MG quantified SA. NFdF, JB and MLdT performed western blots. JC performed kinase assays. SP and SB did the microarray hybridisations. RZ, VB, SA, MLMM and NFdF performed the differential and clustering analysis, prediction of cis-regulatory elements and gene interaction network. NFdF, AVG, JB, EB, MLMM and HH analysed the data. NFdF and AVG wrote the paper with contributions from all authors. All authors read and approved the final manuscript.

## Supplementary Material

Additional file 1: Table S1 List of genes affected in untreated *mpk3*, *mpk4* and *mpk6*or showing differential expression after flg22 treatment. Only genes showing a differential expression (*P* value <0.05) in at least one of the seven comparisons is retained in the table. Complete expression data can be downloaded from CATdb ([91]; Project: RA12-05_mut_flg_II). Click here for file

Additional file 2: Table S2 Differentially expressed genes and GO term enrichment observed in the mock-treated samples from the comparison between Col-0 and *mpk3*, *mpk4* or *mpk6*. Click here for file

Additional file 3: Table S3 Analysis of genes commonly or specifically misregulated in *mpk3* and *mpk4* in mock-treated samples. GO term enrichments associated to the different gene classes are also mentioned. Click here for file

Additional file 4: Figure S1 *mpk3*, *mpk4* and *mpk6* do not mimic the flg22-induced transcriptional reprogramming. (A) Venn diagram of upregulated genes observed in Col-0 after flg22 treatment and in *mpk3*, *mpk4* and *mpk6* in comparison with Col-0. (B) Venn diagram of downregulated genes observed in Col-0 after flg22 treatment and in *mpk3*, *mpk4*and *mpk6* in comparison with Col-0. Note that few genes misregulated in the *MAPK* mutants follow the same misregulation in Col-0 treated with flg22. Click here for file

Additional file 5: Table S4 Differentially expressed genes and GO term enrichment observed in response to flg22 in Col-0, *mpk3*, *mpk4* and *mpk6*. Genes for which the flg22-induced regulation is affected by at least 1 log in *mpk3*, *mpk4* and *mpk6* are mentioned, together with their associated GO term enrichments. Click here for file

Additional file 6: Table S5 Analysis of genes commonly or specifically misregulated in *mpk3*, *mpk4* and *mpk6* after flg22 treatment. GO term enrichments associated to the different gene classes are also mentioned. Click here for file

Additional file 7: Figure S2 Twenty-four percent of the flg22-upregulated *MPK4*-dependent genes are upregulated in mock-treated *mpk4*. (A) Expression profiles of the 89 genes that are upregulated in mock-treated *mpk4* and show reduced flg22-induced upregulation in *mpk4* as compared with Col-0. (B) Expression profiles of the 342 genes that are unmodified in mock-treated *mpk4* and show reduced flg22-induced upregulation in *mpk4* as compared with Col-0. Profiles are represented as boxplots, where the bottom and top of the box are the first and third quartiles and the band inside the box is the median. Data not included between the whiskers are represented by a dot. Click here for file

Additional file 8: Figure S3 Analysis of Gene Ontology (GO) enrichment in flg22-regulated MPK4-dependent genes. (A) Venn diagram analysis of GO families in the two gene groups described in Additional file 7: Figure S2. Numbers inside the Venn diagram correspond to GO categories. (B) Throughout other enrichments, GOs for ethylene signalling and synthesisgenes show MPK4-dependency upon flg22 treatment but not under standard conditions. (C) GOs associated to cell death regulation and immune responses are present in MPK4-dependent genes upon flg22 treatment, but only partially upregulated under standard conditions. (D) GOs related to SA, JA, ROS, cell death and immune responses are present in genes upregulated in *mpk4* in standard conditions, but still show MPK4-dependency upon flg22 treatment. SA: salicylic acid, JA: jasmonic acid, ROS: reactive oxygen species, GO: Gene Ontology, HR: Hypersensitive response, N: number of genes. Note that less genes than previously indicated (Additional file 7: Figure S2) are described here since databases displaying GO enrichment do not contain data for all genes present on CATMA V6.0 chips. Click here for file

Additional file 9: Figure S4 Overview of the clusters obtained from the coexpression analysis. The y-axis shows log ratios. The x-axis shows the following comparisons: Col-0 + flg22 *vs.* Col-0, *mpk3 vs.* Col-0, *mpk4 vs.* Col-0, *mpk6 vs.* Col-0, *mpk3* + flg22 *vs. mpk*3, *mpk4* + flg22 *vs. mpk4*, *mpk6* + flg22 *vs. mpk6*. Profiles are represented as boxplots, where the bottom and top of the box are the first and third quartiles and the band inside the box is the median. Data not included between the whiskers are represented by a dot. On top is indicated the percentage of genes differentially regulated (*P* value <0.05) in the different comparisons. Click here for file

Additional file 10: Table S6 Tables containing the gene lists, ATTED network representations and GO term enrichment for each clusters. Click here for file

Additional file 11: Figure S5 Description of the 10 gene classes defined from the kinetic study performed by Denoux et al. [40]. In the upregulated genes, the discrimination between the classes H, I, J is based on at least a two-fold difference in the fold change observed at 1 h and 3 h. For example, a gene induced 10 times at 1 h and 15 times at 3 h will belong to class H (the difference between 1 h and 3 h is less than two-fold), but a gene induced 10 times at 1 h and induced 50 times at 3 h will belong to class I (the difference in fold change between 1 h and 3 h is greater than 2). Similar analysis is made to build the classes of downregulated genes (Classes A-E). hpt: hours post treatment, a.u.: arbitrary units. Click here for file

Additional file 12: Figure S6 Genes not affected by flg22 in Col-0 after 30 min and upregulated under standard conditions in *mpk4* are enriched in ‘late’ flg22-induced genes. Cluster 4 and 7 do not show enrichment for up- or downregulated genes classes in data from Denoux et al. [40]. Profiles are represented as boxplots, where the bottom and top of the box are the first and third quartiles and the band inside the box is the median. Data not included between the whiskers are represented by a dot. Click here for file

Additional file 13: Figure S7 Flg22-induced *MPK4*-dependent genes are enriched in early and transiently induced genes, as indicated by the comparison with data from Denoux et al. [40]. Profiles are represented as boxplots, where the bottom and top of the box are the first and third quartiles and the band inside the box is the median. Data not included between the whiskers are represented by a dot. Click here for file

Additional file 14: Figure S8 ATTED2 representation of gene co-expression observed in cluster 23. White coloured genes are present in the cluster. Grey coloured genes are out of the cluster but contribute the network. Transcription factors are indicated by octagonal shapes. Coloured dots indicate metabolic pathways. Red: biosynthesis of secondary metabolites (KEGG ID: ath01110), yellow: glucosinolate biosynthesis (KEGG ID: ath00966), green: flavonoid biosynthesis (KEGG ID: ath00941), light blue: glutathione metabolism (KEGG ID: ath00480), blue: valine, leucine and isoleucine biosynthesis (KEGG ID: ath00290). Thickness of lines linking two genes indicates the strength of the co-expression. Orange lines indicate protein-protein interaction. Large circles with dashed lines highlight gene clusters involved in processes of interest for our study. Click here for file

Additional file 15: Figure S9 ATTED2 representation of gene co-expression observed in cluster 16. White coloured genes are present in the cluster, grey coloured genes are outside of the cluster but contribute to the network. Transcription factors are indicated by octagonal shapes. Coloured dots indicate metabolic pathways. Red: valine, leucine and isoleucine degradation (KEGG ID: ath00280), yellow: biosynthesis of secondary metabolites (KEGG ID: ath01110), green: propanoate metabolism (KEGG ID: ath00640), light blue: alanine, aspartate and glutamate metabolism (KEGG ID: ath00250), blue: arginine and proline metabolism (KEGG ID: ath00330). Thickness of lines linking two genes indicates the strength of the co-expression. Orange lines indicate protein-protein interaction. Large circles with dashed lines highlight gene clusters involved in processes of interest for our study. Click here for file

Additional file 16: Figure S10 Clusters 8, 13 and 24 group genes more rapidly regulated by flg22 in *mpk3* and *mpk6*, as indicated by the comparison with ‘late’ flg22-regulated genes (from Denoux et al. [40]). Profiles are represented as boxplots, where the bottom and top of the box are the first and third quartiles and the band inside the box is the median. Data not included between the whiskers are represented by a dot. Click here for file

Additional file 17: Table S7 List of CIS elements enriched in the promoters of the different clusters. Click here for file

Additional file 18: Figure S11 Construction of gene interaction networks. (A) Cytoscape representation of the gene interaction network highlighting the identified regulatory hubs. Zooms into the interaction networks of the regulatory hubs CML9 (B), CPK4 and CPK11 (C), PIF1 (D), HY5 (E). Click here for file

Additional file 19: Figure S12 *mpk3 mpk4* and *mpk6 mpk4* double mutant plants resemble phenotypically single *mpk4* mutant plants. Pictures of 5-week-old soil grown plants of the indicated genotypes. Arrows indicate *mpk4*, *mpk3 mpk4* and *mpk6 mpk4* dwarf plants. Click here for file

Additional file 20: Figure S13 Immunoblot analysis of the protein abundance of MPK3, MPK4 and MPK6 in Col-0, in *mpk3*, *mpk4* and *mpk6* single and in *mpk3 mpk4* and *mpk6 mpk4* double mutants treated with flg22. Western blot analysis of Col-0, *mpk3* and *mpk6* (A) and Col-0, *mpk4*, *mpk3 mpk4* and *mpk6 mpk4* (B) at the indicated time-points after flg22 treatment, using anti-MPK antibodies to detect MPK3, MPK4 and MPK6 abundance. Arrows indicate the protein bands corresponding to MPK3, MPK4 and MPK6. The size of the molecular weight (MW) markers is indicated in kDa on the left. Blots were stained with Coomassie blue for protein visualization; the lower panels in A and B show the protein band corresponding to the RuBisCO large subunit. Click here for file

Additional file 21: Figure S14 MPK3, MPK4 and MPK6 do not phosphorylate each other in *in vitro* kinase assays. (A) Kinase activity of recombinant wild type and constitutive active (Y and DE variants) MPK3, MPK4 and MPK6 towards MBP. (B) Kinase activity of recombinant wild type and constitutive active MPK3, MPK4 and MPK6 towards kinase dead MPK3 and MPK6 variants fused to GST. Upper panels indicate kinase activities (autoradiographs) and lower panels show Coomassie blue staining of the gels to indicate equal loading. Upper panels indicate kinase activities (autoradiographs) and lower panels show Coomassie blue staining of the gels to indicate equal loading. Click here for file
