# Supplementary figures and images for: Functional analysis of Arabidopsis immune-related MAPKs uncovers a role for MPK3 as negative regulator of inducible defences
Source: Genome Biol. 2014 Jun 30;15(6):R87. doi: 10.1186/gb-2014-15-6-r87 (PMC4197828; doi:10.1186/gb-2014-15-6-r87)

**Figure S1**

**A**

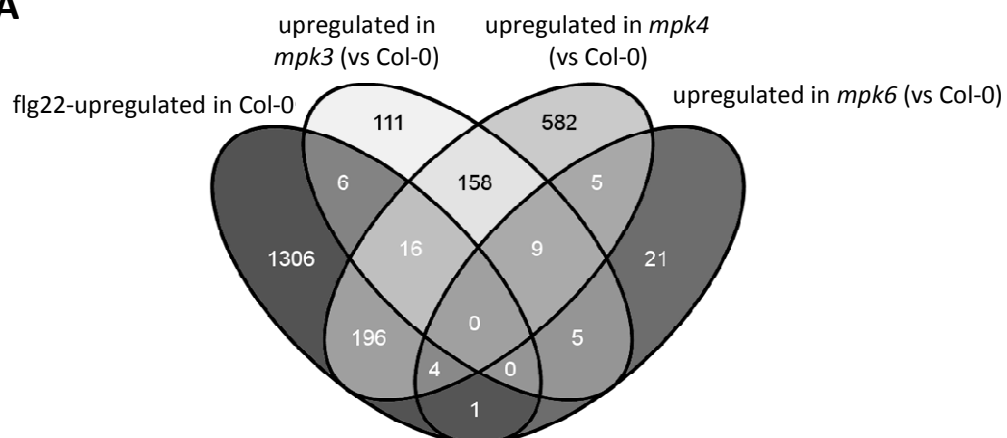

**B**

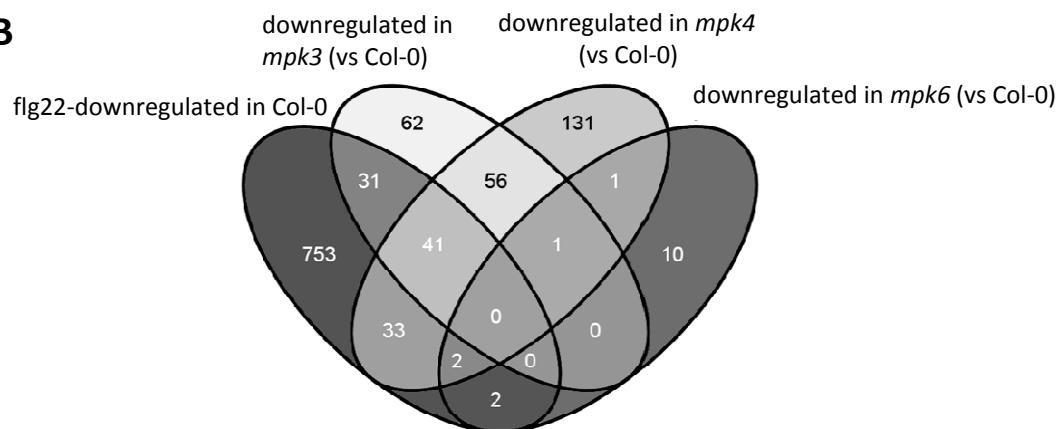

Supplement: Additional file 4: Figure S1 — mpk3, mpk4 and mpk6 do not mimic the flg22-induced transcriptional reprogramming. (A) Venn diagram of upregulated genes observed in Col-0 after flg22 treatment and in mpk3, mpk4 and mpk6 in comparison with Col-0. (B) Venn diagram of downregulated genes observed in Col-0 after flg22 treatment and in mpk3, mpk4and mpk6 in comparison with Col-0. Note that few genes misregulated in the MAPK mutants follow the same misregulation in Col-0 treated with flg22. [file gb-2014-15-6-r87-S4.pdf]

**Figure S1**

**A**

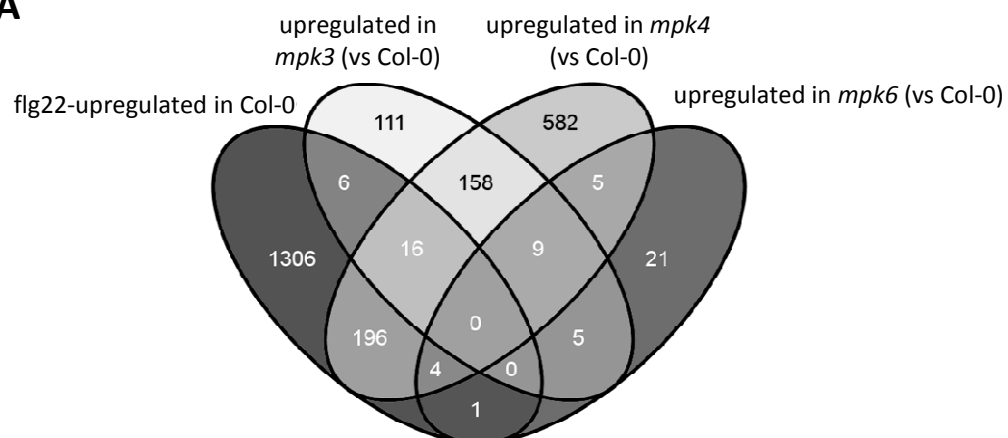

**B**

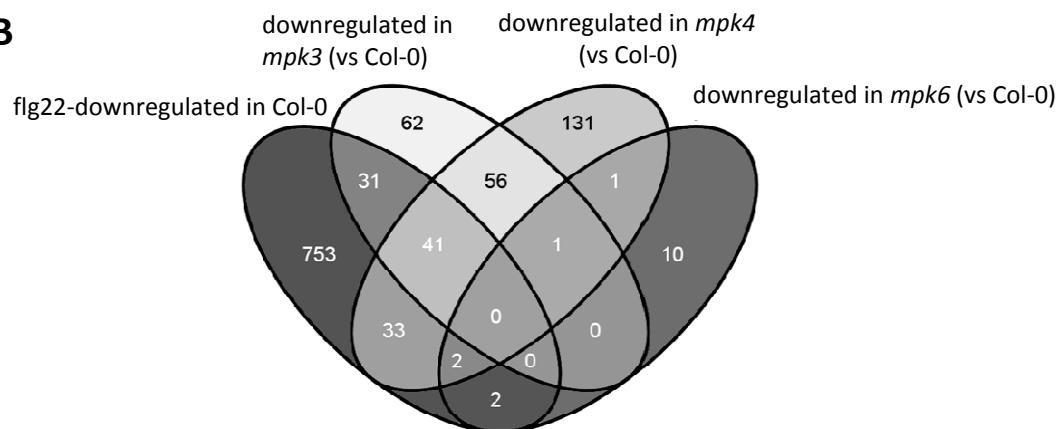

Supplement: Additional file 7: Figure S2 — Twenty-four percent of the flg22-upregulated MPK4-dependent genes are upregulated in mock-treated mpk4. (A) Expression profiles of the 89 genes that are upregulated in mock-treated mpk4 and show reduced flg22-induced upregulation in mpk4 as compared with Col-0. (B) Expression profiles of the 342 genes that are unmodified in mock-treated mpk4 and show reduced flg22-induced upregulation in mpk4 as compared with Col-0. Profiles are represented as boxplots, where the bottom and top of the box are the first and third quartiles and the band inside the box is the median. Data not included between the whiskers are represented by a dot. [file gb-2014-15-6-r87-S7.pdf]

**Figure S5**

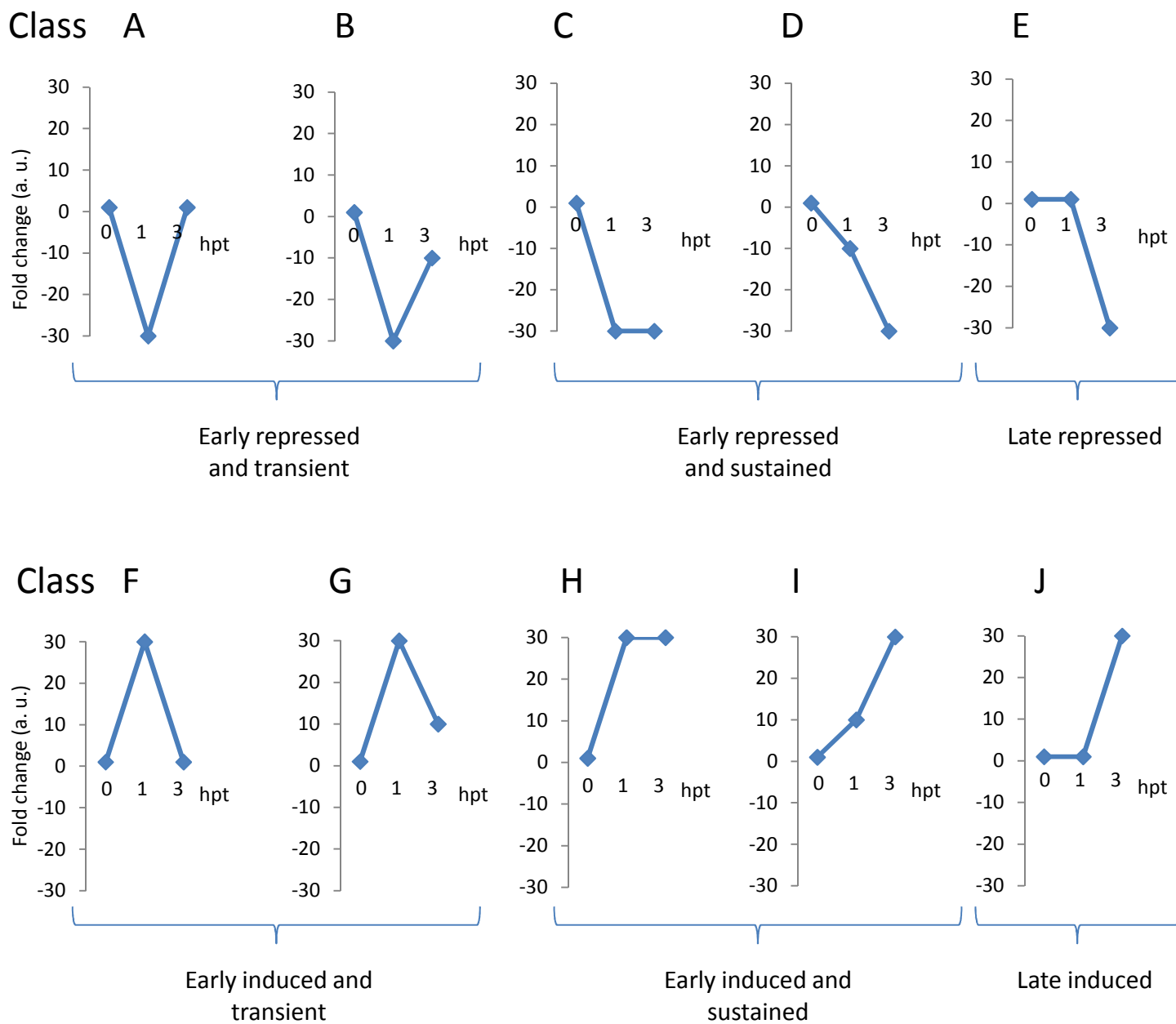

Supplement: Additional file 11: Figure S5 — Description of the 10 gene classes defined from the kinetic study performed by Denoux et al. [40]. In the upregulated genes, the discrimination between the classes H, I, J is based on at least a two-fold difference in the fold change observed at 1 h and 3 h. For example, a gene induced 10 times at 1 h and 15 times at 3 h will belong to class H (the difference between 1 h and 3 h is less than two-fold), but a gene induced 10 times at 1 h and induced 50 times at 3 h will belong to class I (the difference in fold change between 1 h and 3 h is greater than 2). Similar analysis is made to build the classes of downregulated genes (Classes A-E). hpt: hours post treatment, a.u.: arbitrary units. [file gb-2014-15-6-r87-S11.pdf]

**Figure S6**

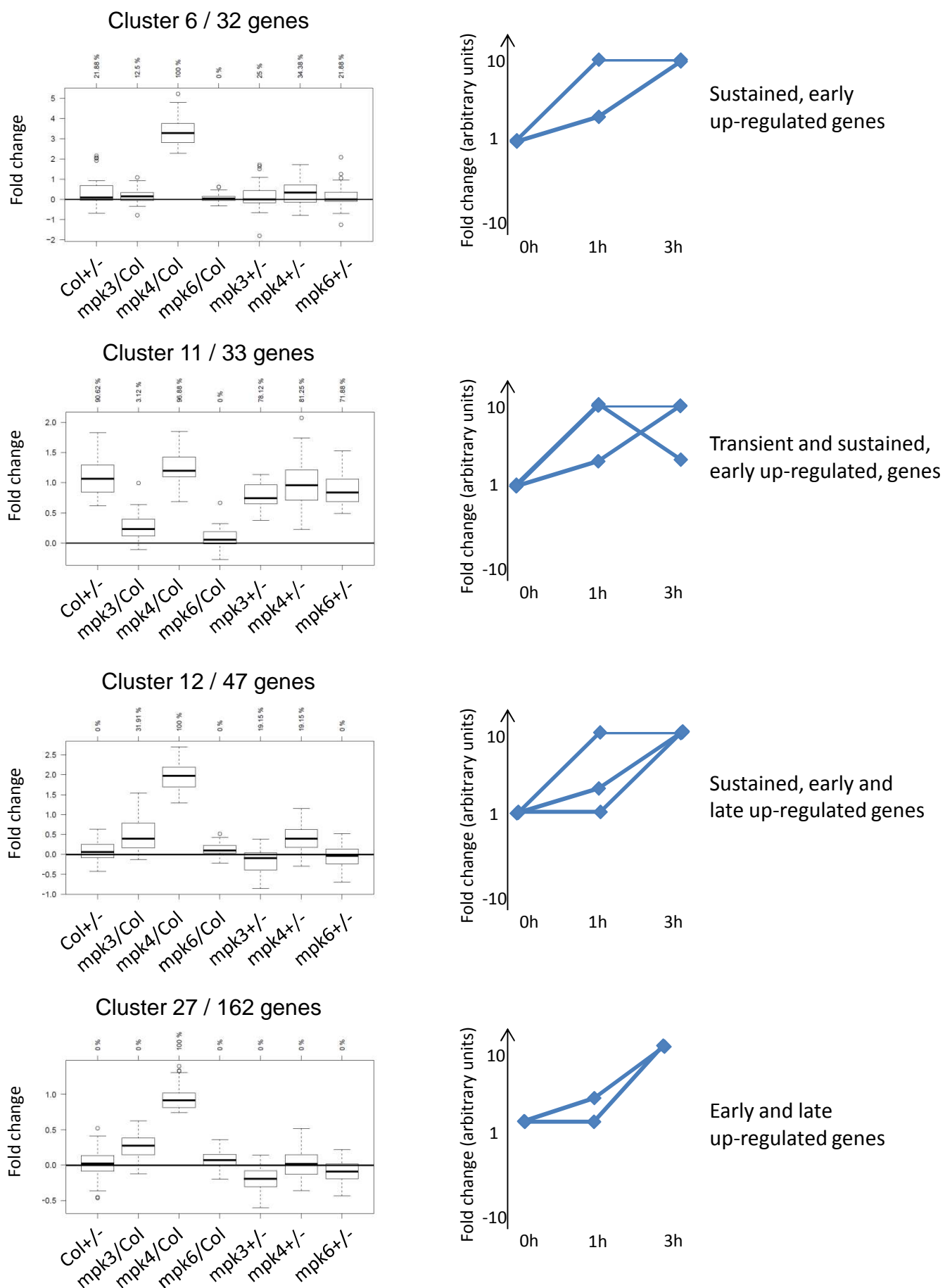

Supplement: Additional file 12: Figure S6 — Genes not affected by flg22 in Col-0 after 30 min and upregulated under standard conditions in mpk4 are enriched in ‘late’ flg22-induced genes. Cluster 4 and 7 do not show enrichment for up- or downregulated genes classes in data from Denoux et al. [40]. Profiles are represented as boxplots, where the bottom and top of the box are the first and third quartiles and the band inside the box is the median. Data not included between the whiskers are represented by a dot. [file gb-2014-15-6-r87-S12.pdf]

Figure S7

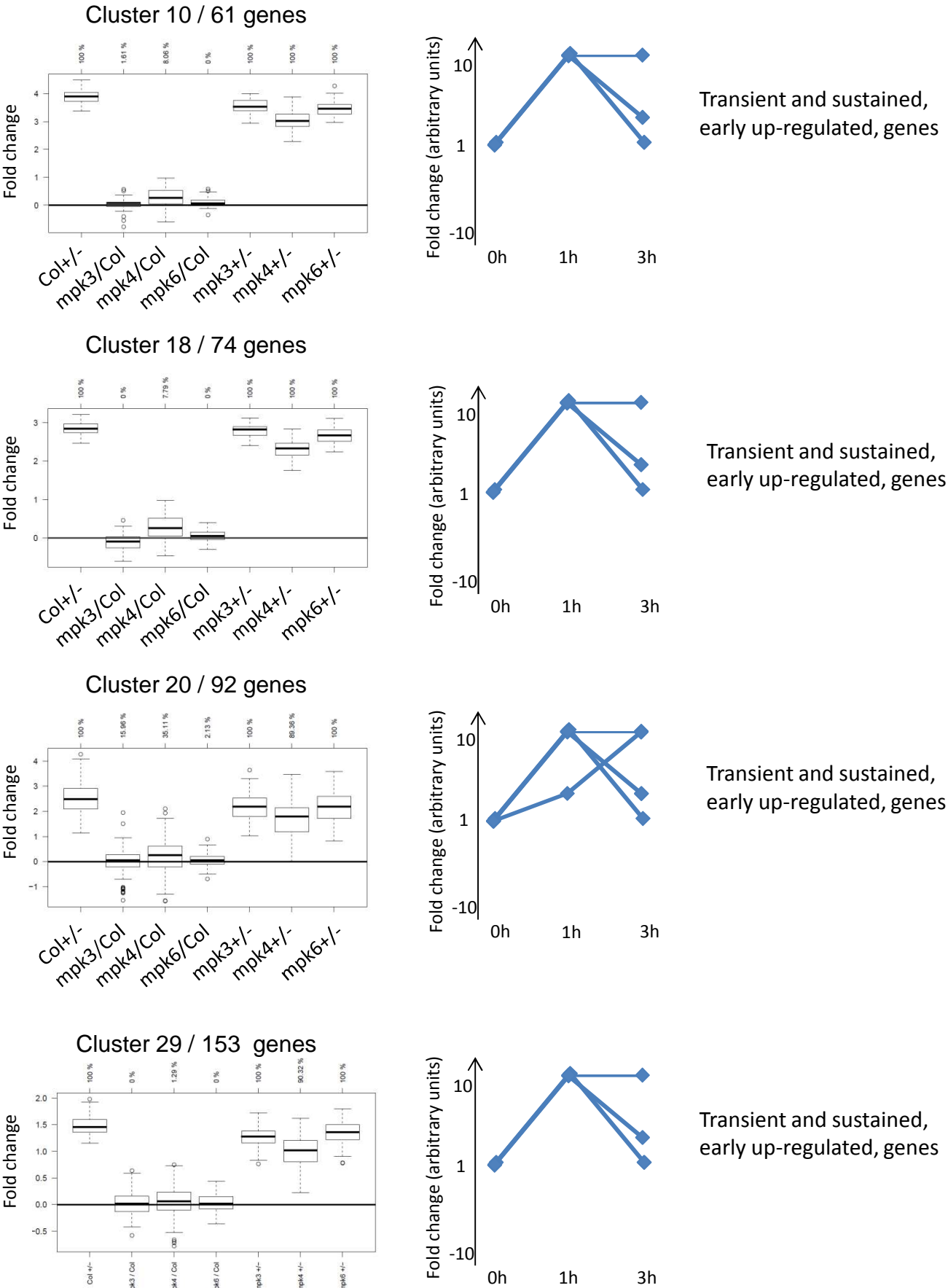

Supplement: Additional file 13: Figure S7 — Flg22-induced MPK4-dependent genes are enriched in early and transiently induced genes, as indicated by the comparison with data from Denoux et al. [40]. Profiles are represented as boxplots, where the bottom and top of the box are the first and third quartiles and the band inside the box is the median. Data not included between the whiskers are represented by a dot. [file gb-2014-15-6-r87-S13.pdf]

### Cluster 23

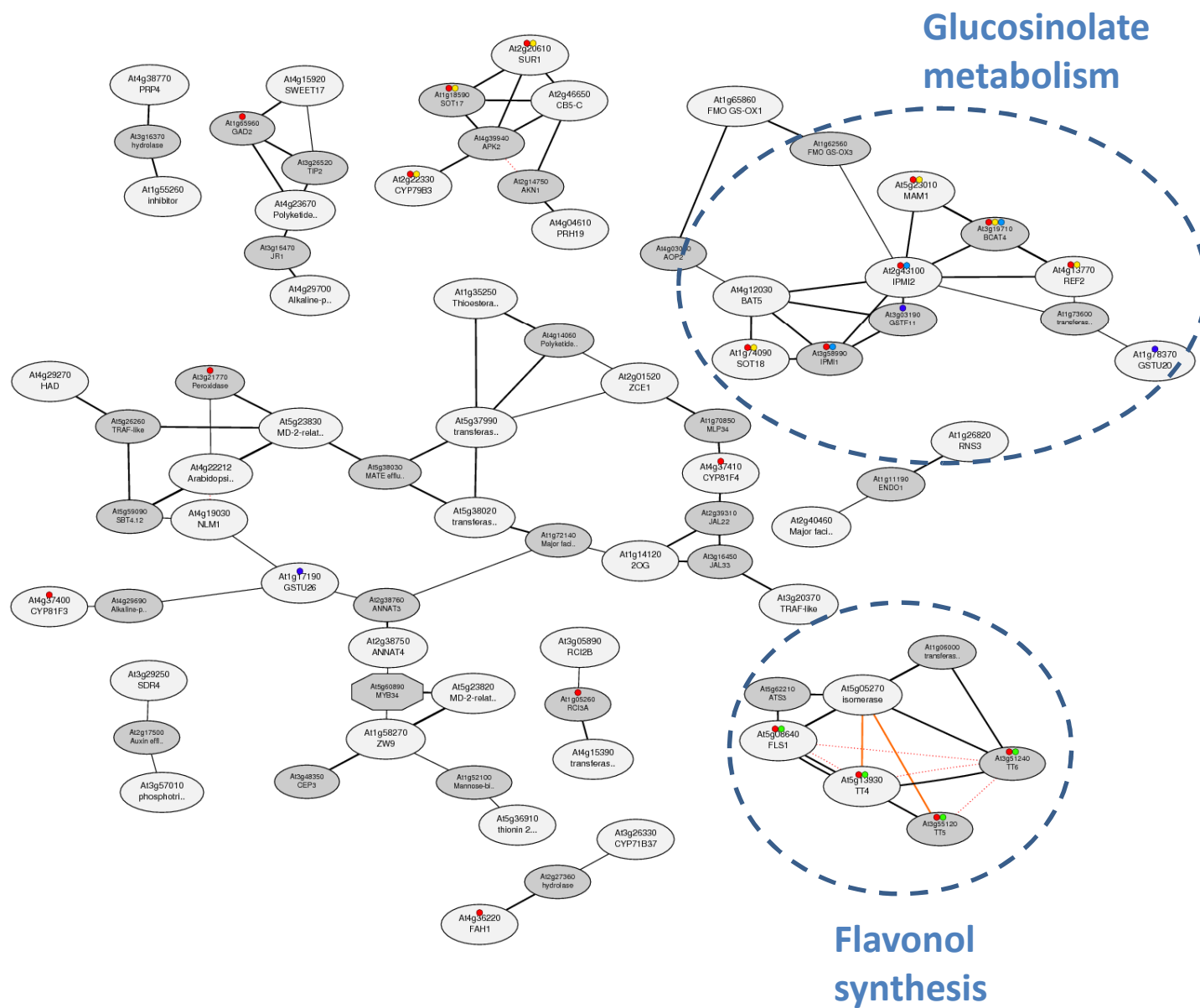

Supplement: Additional file 14: Figure S8 — ATTED2 representation of gene co-expression observed in cluster 23. White coloured genes are present in the cluster. Grey coloured genes are out of the cluster but contribute the network. Transcription factors are indicated by octagonal shapes. Coloured dots indicate metabolic pathways. Red: biosynthesis of secondary metabolites (KEGG ID: ath01110), yellow: glucosinolate biosynthesis (KEGG ID: ath00966), green: flavonoid biosynthesis (KEGG ID: ath00941), light blue: glutathione metabolism (KEGG ID: ath00480), blue: valine, leucine and isoleucine biosynthesis (KEGG ID: ath00290). Thickness of lines linking two genes indicates the strength of the co-expression. Orange lines indicate protein-protein interaction. Large circles with dashed lines highlight gene clusters involved in processes of interest for our study. [file gb-2014-15-6-r87-S14.pdf]

Figure S9

Cluster 16

Branched-Chain  
Amino Acid  
degradation

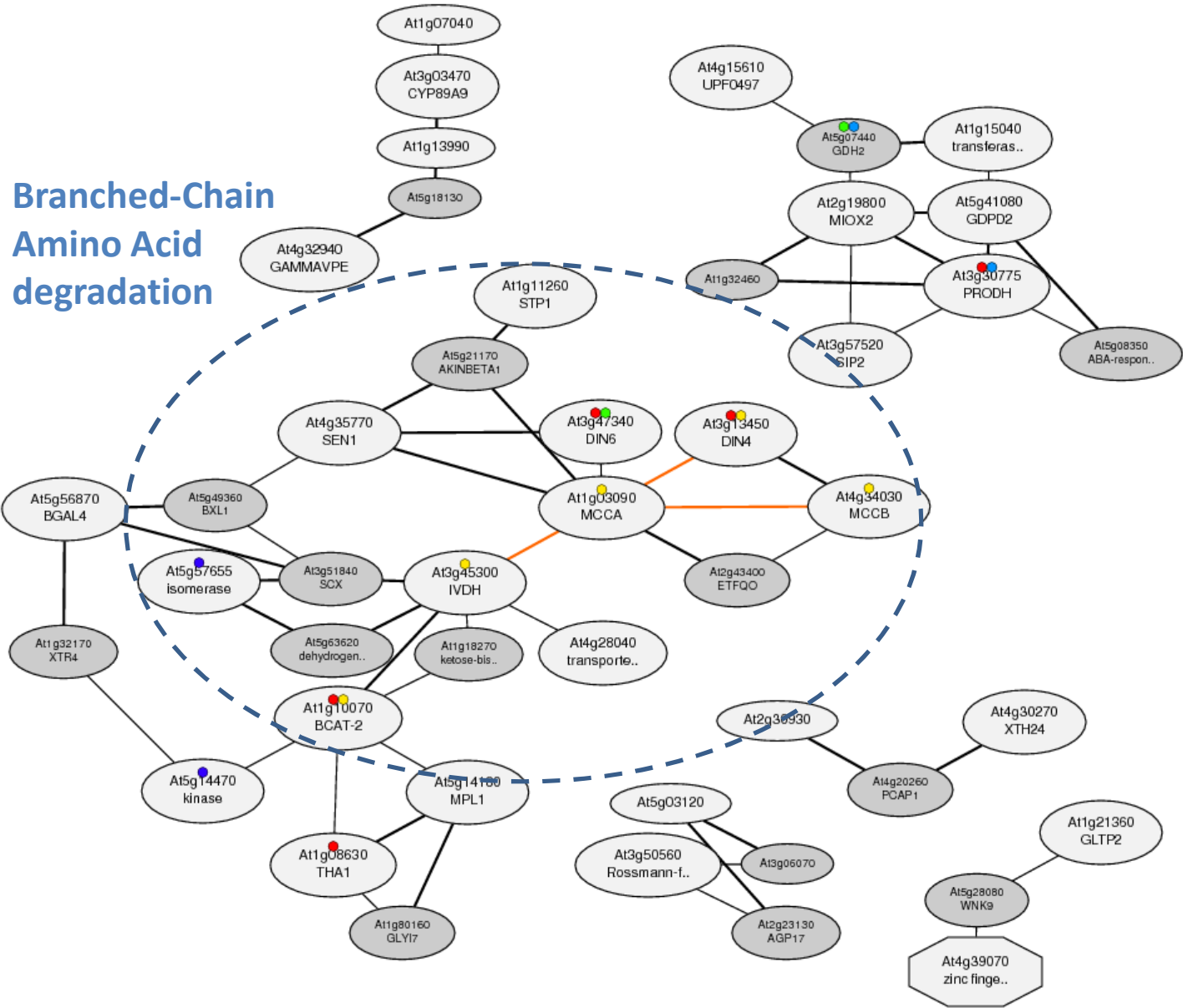

Supplement: Additional file 15: Figure S9 — ATTED2 representation of gene co-expression observed in cluster 16. White coloured genes are present in the cluster, grey coloured genes are outside of the cluster but contribute to the network. Transcription factors are indicated by octagonal shapes. Coloured dots indicate metabolic pathways. Red: valine, leucine and isoleucine degradation (KEGG ID: ath00280), yellow: biosynthesis of secondary metabolites (KEGG ID: ath01110), green: propanoate metabolism (KEGG ID: ath00640), light blue: alanine, aspartate and glutamate metabolism (KEGG ID: ath00250), blue: arginine and proline metabolism (KEGG ID: ath00330). Thickness of lines linking two genes indicates the strength of the co-expression. Orange lines indicate protein-protein interaction. Large circles with dashed lines highlight gene clusters involved in processes of interest for our study. [file gb-2014-15-6-r87-S15.pdf]

Figure S12

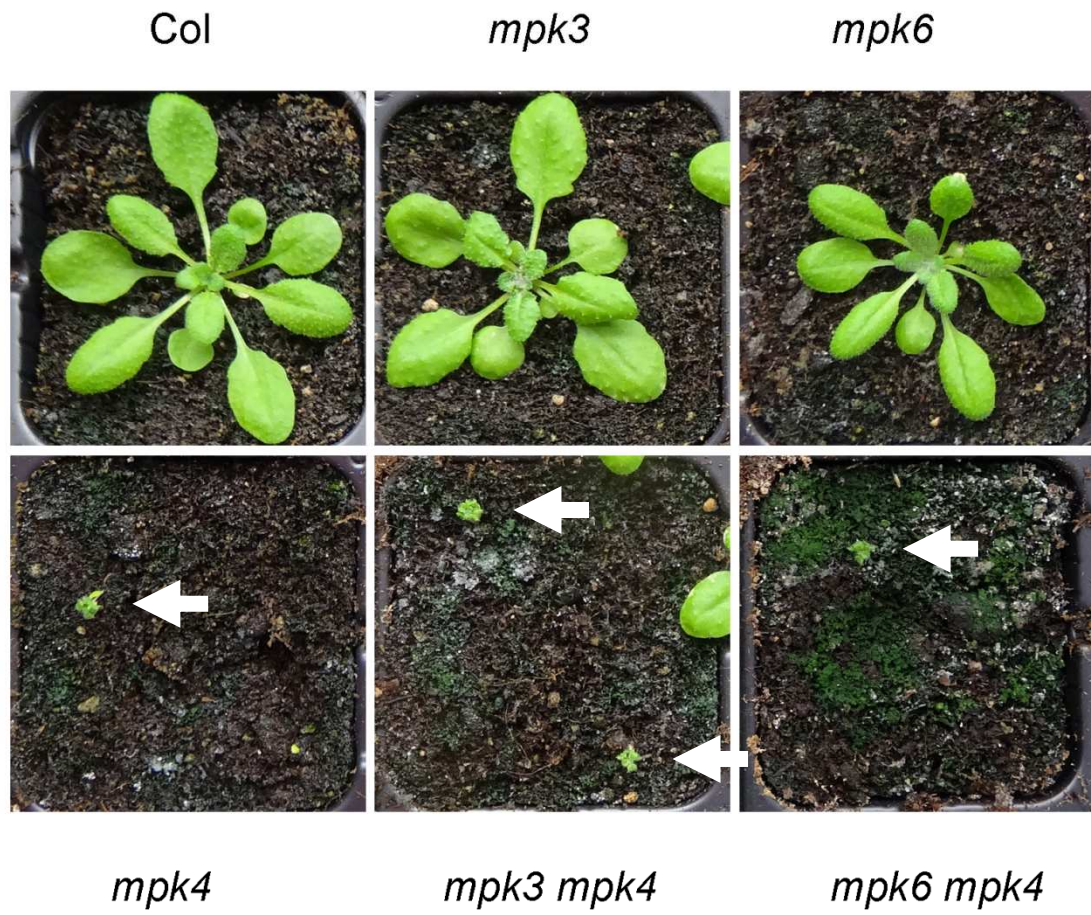

Supplement: Additional file 19: Figure S12 — mpk3 mpk4 and mpk6 mpk4 double mutant plants resemble phenotypically single mpk4 mutant plants. Pictures of 5-week-old soil grown plants of the indicated genotypes. Arrows indicate mpk4, mpk3 mpk4 and mpk6 mpk4 dwarf plants. [file gb-2014-15-6-r87-S19.pdf]

### Figure S13

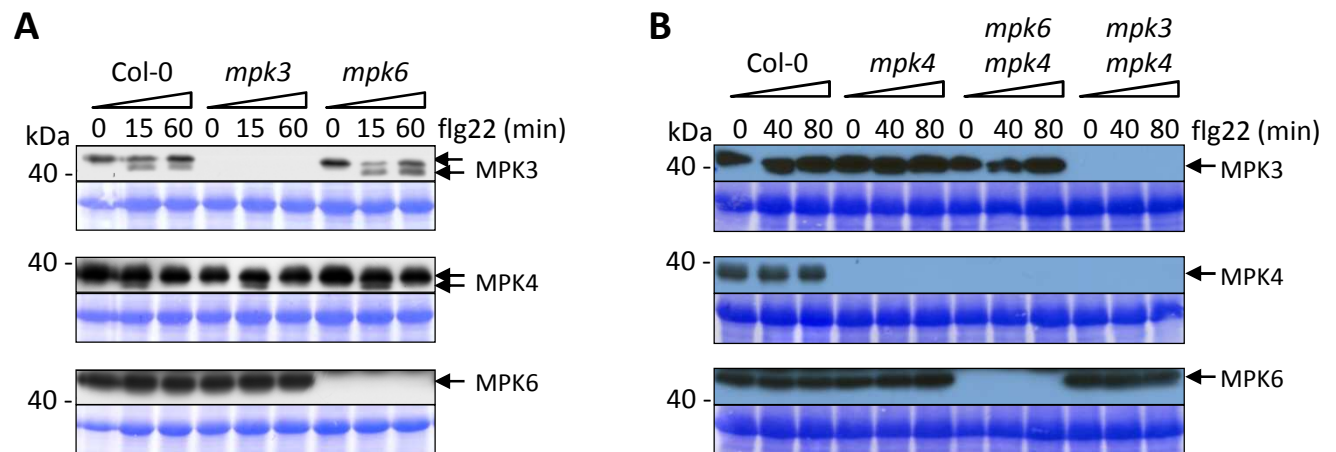

Supplement: Additional file 20: Figure S13 — Immunoblot analysis of the protein abundance of MPK3, MPK4 and MPK6 in Col-0, in mpk3, mpk4 and mpk6 single and in mpk3 mpk4 and mpk6 mpk4 double mutants treated with flg22. Western blot analysis of Col-0, mpk3 and mpk6 (A) and Col-0, mpk4, mpk3 mpk4 and mpk6 mpk4 (B) at the indicated time-points after flg22 treatment, using anti-MPK antibodies to detect MPK3, MPK4 and MPK6 abundance. Arrows indicate the protein bands corresponding to MPK3, MPK4 and MPK6. The size of the molecular weight (MW) markers is indicated in kDa on the left. Blots were stained with Coomassie blue for protein visualization; the lower panels in A and B show the protein band corresponding to the RuBisCO large subunit. [file gb-2014-15-6-r87-S20.pdf]

Figure S14

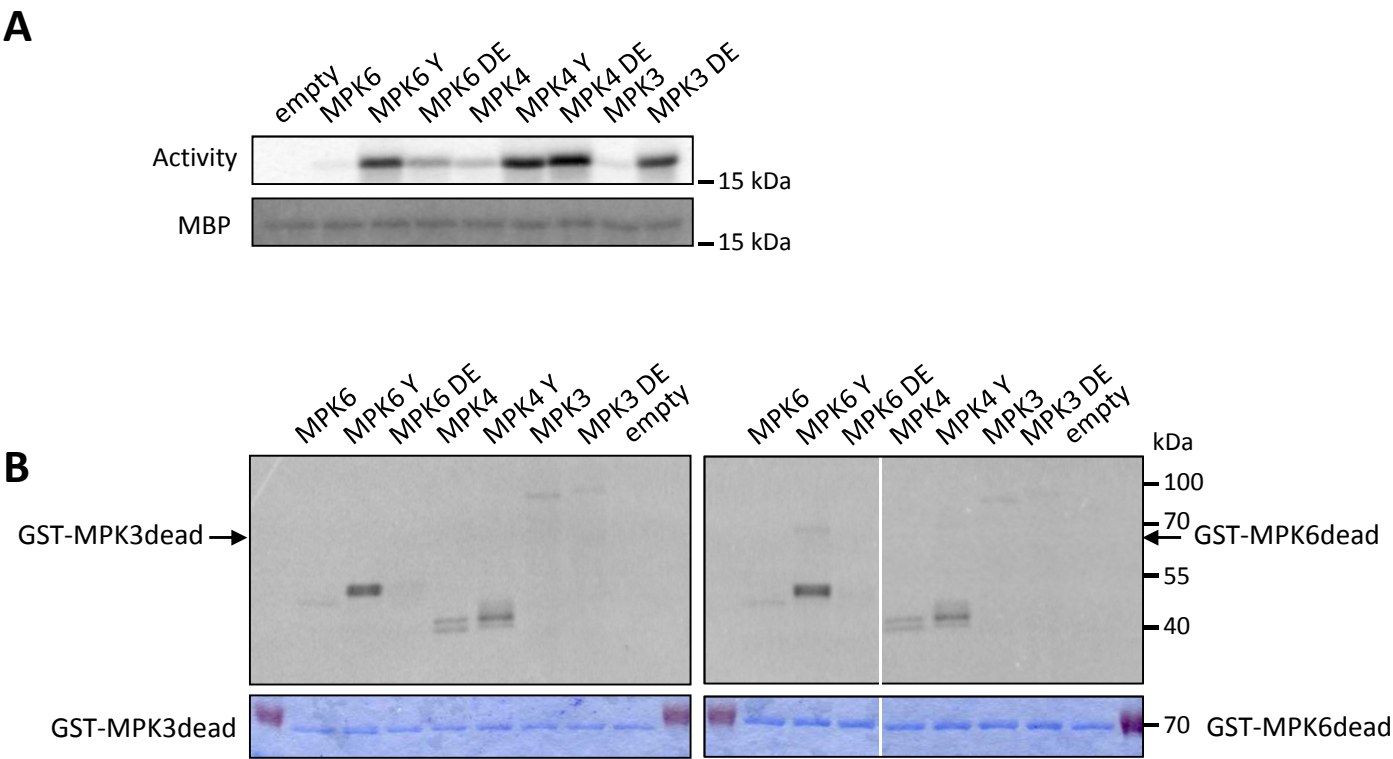

Supplement: Additional file 21: Figure S14 — MPK3, MPK4 and MPK6 do not phosphorylate each other in in vitro kinase assays. (A) Kinase activity of recombinant wild type and constitutive active (Y and DE variants) MPK3, MPK4 and MPK6 towards MBP. (B) Kinase activity of recombinant wild type and constitutive active MPK3, MPK4 and MPK6 towards kinase dead MPK3 and MPK6 variants fused to GST. Upper panels indicate kinase activities (autoradiographs) and lower panels show Coomassie blue staining of the gels to indicate equal loading. Upper panels indicate kinase activities (autoradiographs) and lower panels show Coomassie blue staining of the gels to indicate equal loading. [file gb-2014-15-6-r87-S21.pdf]
