# Supplementary material for: Functional analysis of Arabidopsis immune-related MAPKs uncovers a role for MPK3 as negative regulator of inducible defences
Source: Genome Biol. 2014 Jun 30;15(6):R87. doi: 10.1186/gb-2014-15-6-r87 (PMC4197828; doi:10.1186/gb-2014-15-6-r87)
Supplement: Additional file 8: Figure S3 — Analysis of Gene Ontology (GO) enrichment in flg22-regulated MPK4-dependent genes. (A) Venn diagram analysis of GO families in the two gene groups described in Additional file 7: Figure S2. Numbers inside the Venn diagram correspond to GO categories. (B) Throughout other enrichments, GOs for ethylene signalling and synthesisgenes show MPK4-dependency upon flg22 treatment but not under standard conditions. (C) GOs associated to cell death regulation and immune responses are present in MPK4-dependent genes upon flg22 treatment, but only partially upregulated under standard conditions. (D) GOs related to SA, JA, ROS, cell death and immune responses are present in genes upregulated in mpk4 in standard conditions, but still show MPK4-dependency upon flg22 treatment. SA: salicylic acid, JA: jasmonic acid, ROS: reactive oxygen species, GO: Gene Ontology, HR: Hypersensitive response, N: number of genes. Note that less genes than previously indicated (Additional file 7: Figure S2) are described here since databases displaying GO enrichment do not contain data for all genes present on CATMA V6.0 chips. [file gb-2014-15-6-r87-S8.pdf]

Figure S3

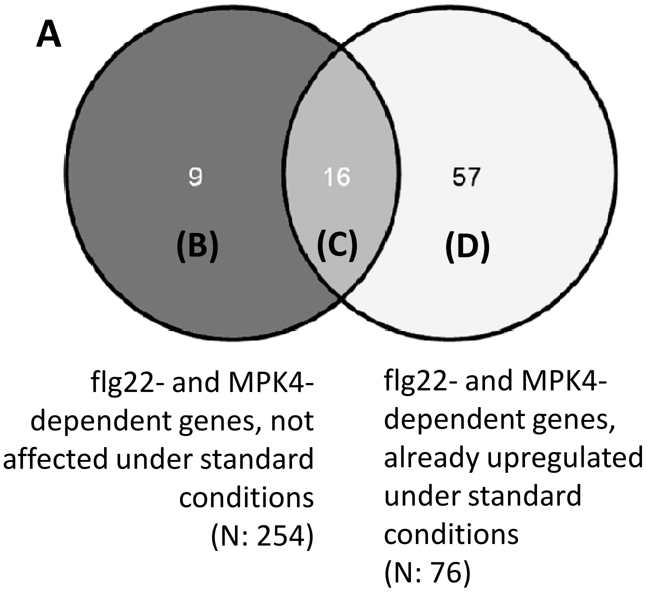

B

GO:1900674 olefin biosynthetic process
GO:0043449 cellular alkene metabolic process
GO:0009692 ethylene metabolic process
GO:0043450 alkene biosynthetic process
GO:1900673 olefin metabolic process
GO:0009693 ethylene biosynthetic process
GO:0010033 response to organic substance
GO:0016998 cell wall macromolecule catabolic process
GO:0042221 response to chemical stimulus

C

GO:0010200 response to chitin
GO:0010243 response to organic nitrogen
GO:0009719 response to endogenous stimulus
GO:0002376 immune system process
GO:0050896 response to stimulus
GO:0006952 defense response
GO:0050832 defense response to fungus
GO:0080135 regulation of cellular response to stress
GO:0009620 response to fungus
GO:0009626 plant-type hypersensitive response
GO:0034050 host programmed cell death induced by symbiont
GO:0010363 regulation of plant-type hypersensitive response
GO:0006612 protein targeting to membrane
GO:0016265 death
GO:0008219 cell death
GO:0045087 innate immune response

D

GO:0051707 response to other organism
GO:0009607 response to biotic stimulus
GO:0009697 salicylic acid biosynthetic process
GO:0009696 salicylic acid metabolic process
GO:0006955 immune response
GO:0042537 benzene-containing compound metabolic process
GO:0072330 monocarboxylic acid biosynthetic process
GO:0009814 defense response, incompatible interaction
GO:0009627 systemic acquired resistance
GO:0016053 organic acid biosynthetic process
GO:0046394 carboxylic acid biosynthetic process
GO:0031347 regulation of defense response
GO:0080134 regulation of response to stress
GO:0032787 monocarboxylic acid metabolic process
GO:0044283 small molecule biosynthetic process
GO:0044711 single-organism biosynthetic process
GO:0009617 response to bacterium
GO:0048583 regulation of response to stimulus
GO:0045088 regulation of innate immune response
GO:0050776 regulation of immune response
GO:0002682 regulation of immune system process
GO:0019752 carboxylic acid metabolic process
GO:0006950 response to stress
GO:0043436 oxoacid metabolic process
GO:0006082 organic acid metabolic process
GO:0009863 salicylic acid mediated signaling pathway
GO:0071446 cellular response to salicylic acid stimulus
GO:0031348 negative regulation of defense response
GO:0000165 MAPK cascade
GO:0007243 intracellular protein kinase cascade
GO:0048585 negative regulation of response to stimulus
GO:0009753 response to jasmonic acid stimulus
GO:0009751 response to salicylic acid stimulus
GO:0042742 defense response to bacterium
GO:0051704 multi-organism process
GO:0071407 cellular response to organic cyclic compound
GO:0012501 programmed cell death
GO:0043069 negative regulation of programmed cell death
GO:0060548 negative regulation of cell death
GO:0010310 regulation of hydrogen peroxide metabolic process
GO:0009867 jasmonic acid mediated signaling pathway
GO:0071395 cellular response to jasmonic acid stimulus
GO:0043067 regulation of programmed cell death
GO:2000377 regulation of reactive oxygen species metabolic process
GO:0010941 regulation of cell death
GO:0014070 response to organic cyclic compound
GO:0070887 cellular response to chemical stimulus
GO:0035556 intracellular signal transduction
GO:0044281 small molecule metabolic process
GO:0072593 reactive oxygen species metabolic process
GO:0009862 SAR, SAmediated signaling pathway
GO:0002237 response to molecule of bacterial origin
GO:0007165 signal transduction
GO:0009595 detection of biotic stimulus
GO:0071310 cellular response to organic substance
GO:0044700 single organism signaling
GO:0023052 signaling
