# Supplementary material for: Functional analysis of Arabidopsis immune-related MAPKs uncovers a role for MPK3 as negative regulator of inducible defences
Source: Genome Biol. 2014 Jun 30;15(6):R87. doi: 10.1186/gb-2014-15-6-r87 (PMC4197828; doi:10.1186/gb-2014-15-6-r87)

Figure S4

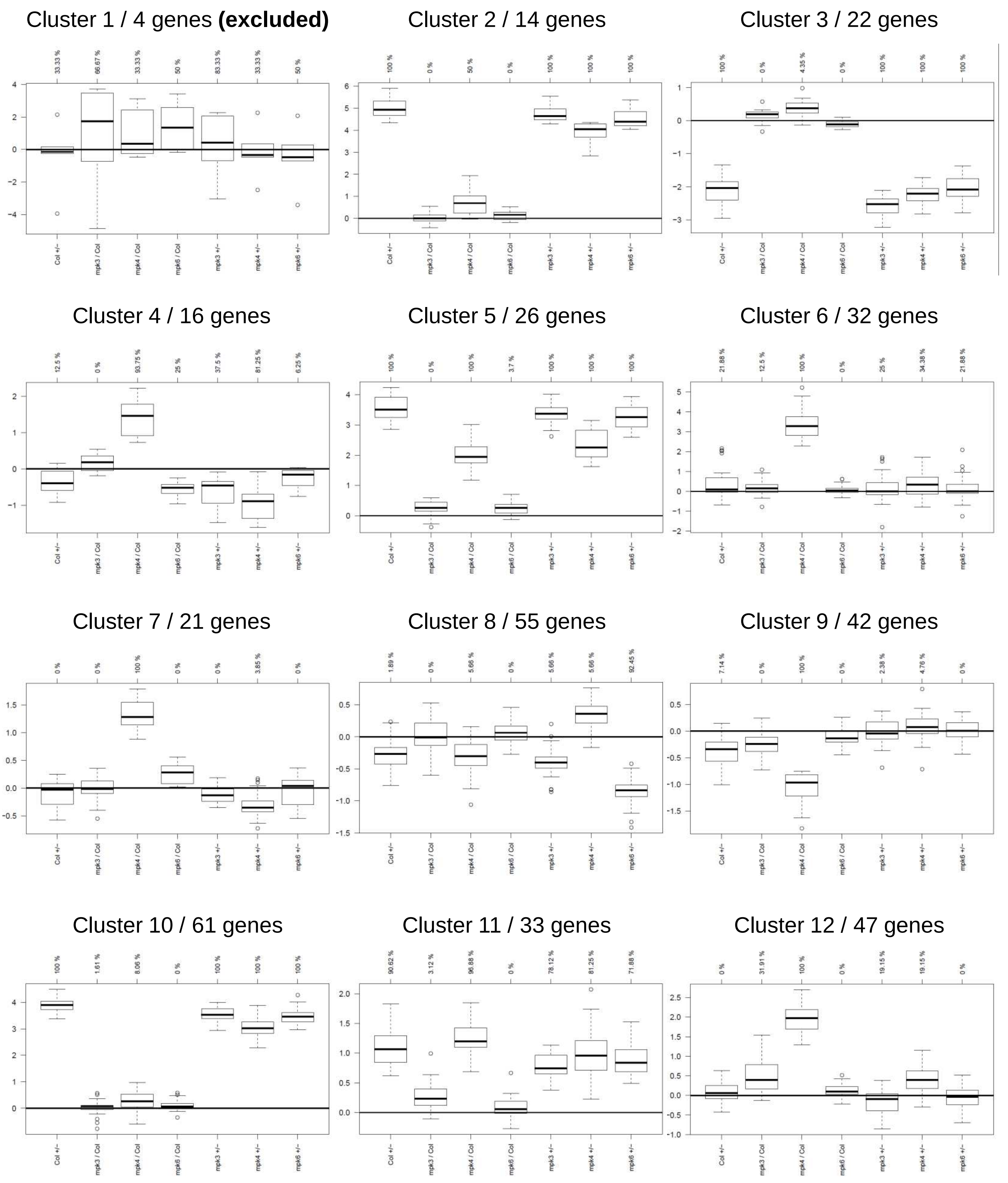
Cluster 1 / 4 genes (excluded)
Cluster 2 / 14 genes
Cluster 3 / 22 genes
Cluster 4 / 16 genes
Cluster 5 / 26 genes
Cluster 6 / 32 genes
Cluster 7 / 21 genes
Cluster 8 / 55 genes
Cluster 9 / 42 genes
Cluster 10 / 61 genes
Cluster 11 / 33 genes
Cluster 12 / 47 genes
Col +/-
mpk3 / Col
mpk4 / Col
mpk6 / Col
mpk3 +/-
mpk4 +/-
mpk6 +/-

Cluster 13 / 38 genes

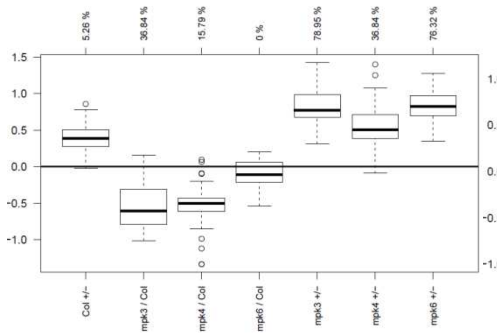

Cluster 14 / 55 genes

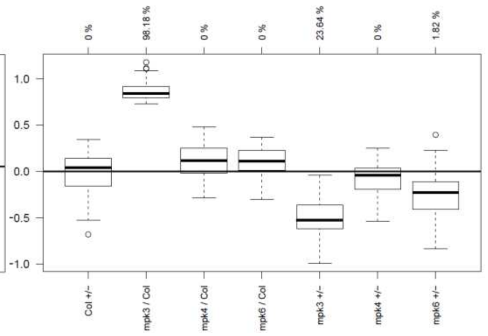

Cluster 15 / 58 genes

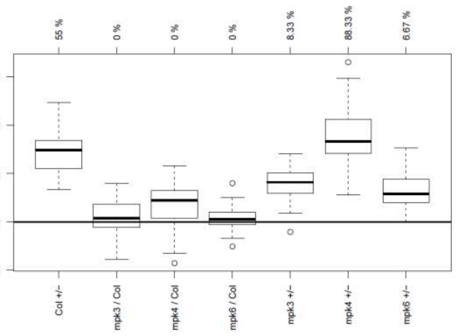

Cluster 16 / 74 genes

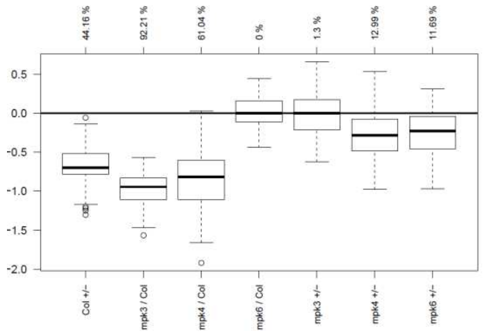

Cluster 17 / 84 genes **(excluded)**

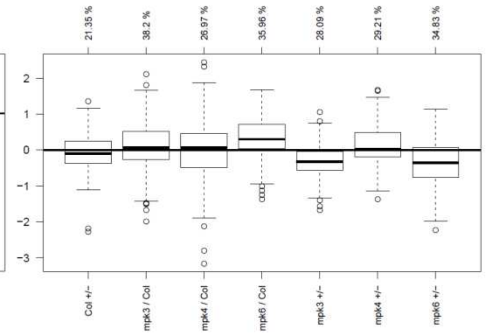

Cluster 18 / 74 genes

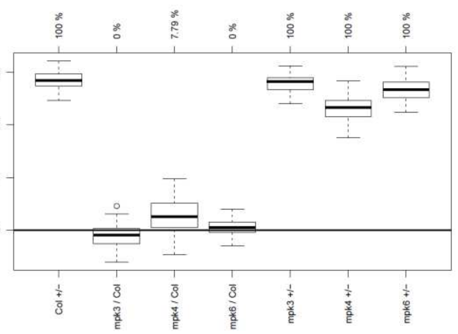

Cluster 19 / 109 genes

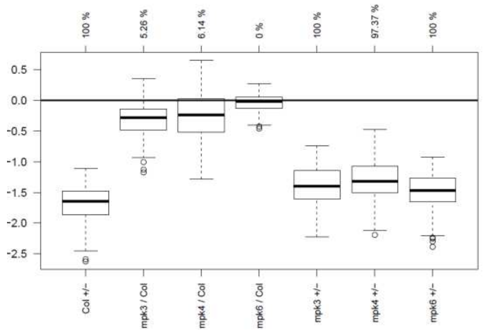

Cluster 20 / 92 genes

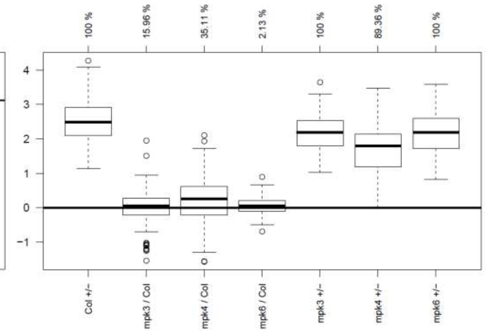

Cluster 21 / 63 genes

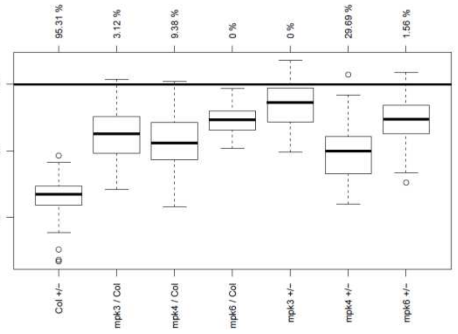

Cluster 22 / 66 genes

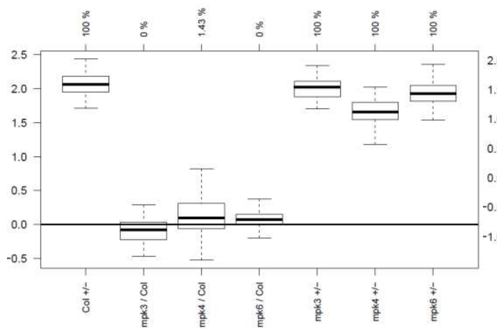

Cluster 23 / 111 genes

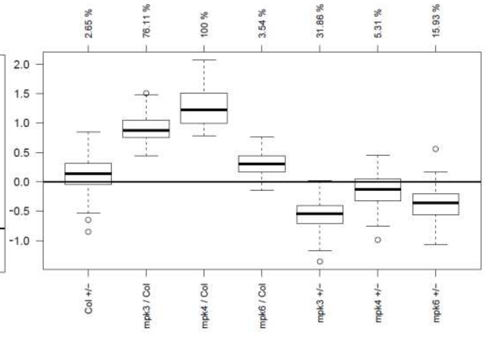

Cluster 24 / 91 genes

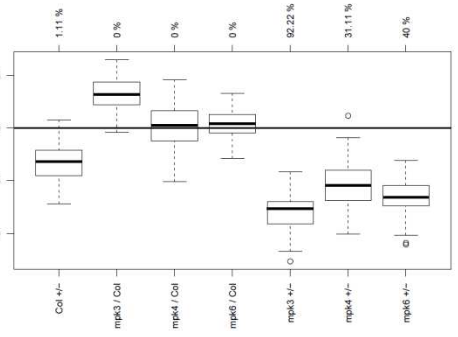

Cluster 25 / 81 genes

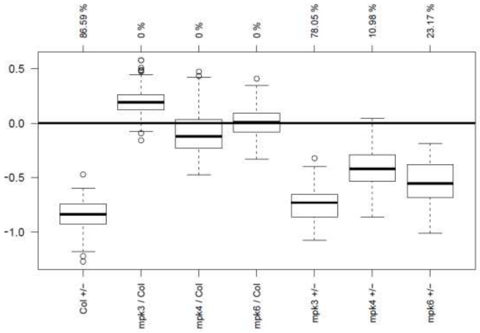

Cluster 26 / 56 genes

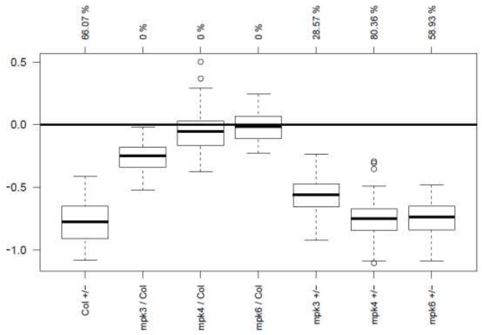

Cluster 27 / 162 genes

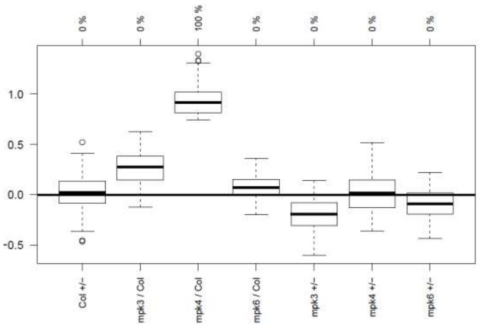

Cluster 28 / 146 genes

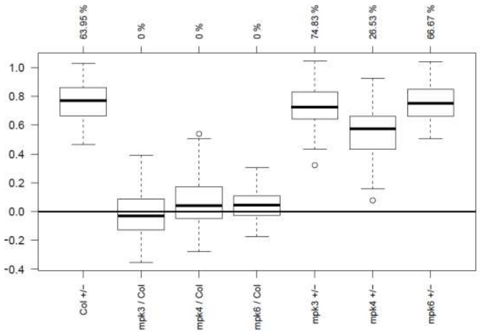

Cluster 29 / 153 genes

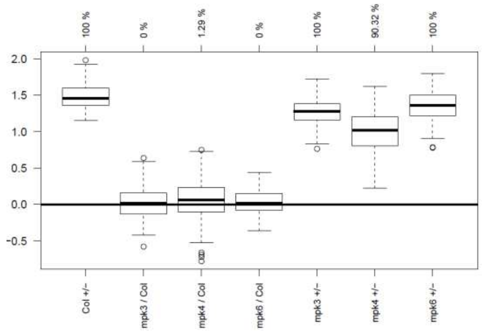

Supplement: Additional file 9: Figure S4 — Overview of the clusters obtained from the coexpression analysis. The y-axis shows log ratios. The x-axis shows the following comparisons: Col-0 + flg22 vs. Col-0, mpk3 vs. Col-0, mpk4 vs. Col-0, mpk6 vs. Col-0, mpk3 + flg22 vs. mpk3, mpk4 + flg22 vs. mpk4, mpk6 + flg22 vs. mpk6. Profiles are represented as boxplots, where the bottom and top of the box are the first and third quartiles and the band inside the box is the median. Data not included between the whiskers are represented by a dot. On top is indicated the percentage of genes differentially regulated (P value <0.05) in the different comparisons. [file gb-2014-15-6-r87-S9.pdf]
