# Supplementary material for: Functional analysis of Arabidopsis immune-related MAPKs uncovers a role for MPK3 as negative regulator of inducible defences
Source: Genome Biol. 2014 Jun 30;15(6):R87. doi: 10.1186/gb-2014-15-6-r87 (PMC4197828; doi:10.1186/gb-2014-15-6-r87)
Supplement: Additional file 16: Figure S10 — Clusters 8, 13 and 24 group genes more rapidly regulated by flg22 in mpk3 and mpk6, as indicated by the comparison with ‘late’ flg22-regulated genes (from Denoux et al. [40]). Profiles are represented as boxplots, where the bottom and top of the box are the first and third quartiles and the band inside the box is the median. Data not included between the whiskers are represented by a dot. [file gb-2014-15-6-r87-S16.pdf]

Figure S10

Genes downregulated only in flg22-treated *mpk6*

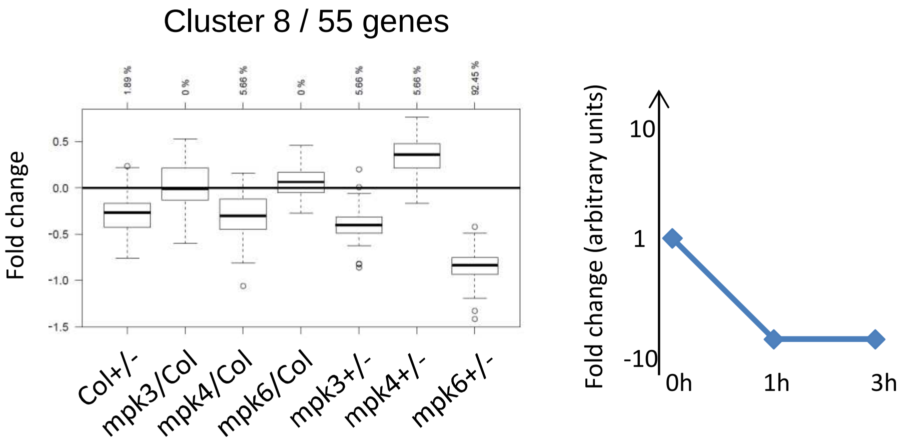

Early and sustained down-regulated genes

Genes upregulated only in flg22-treated *mpk3* and *mpk6*

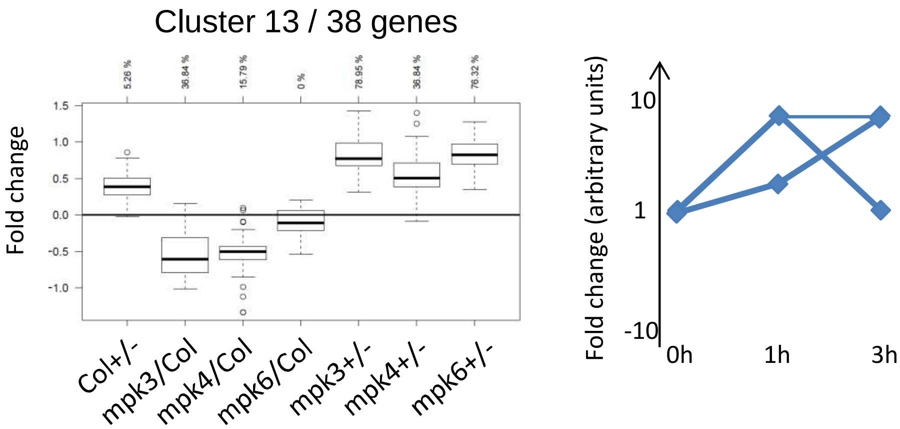

Early , transient and sustained up-regulated genes

Genes downregulated only in flg22-treated *mpk3*

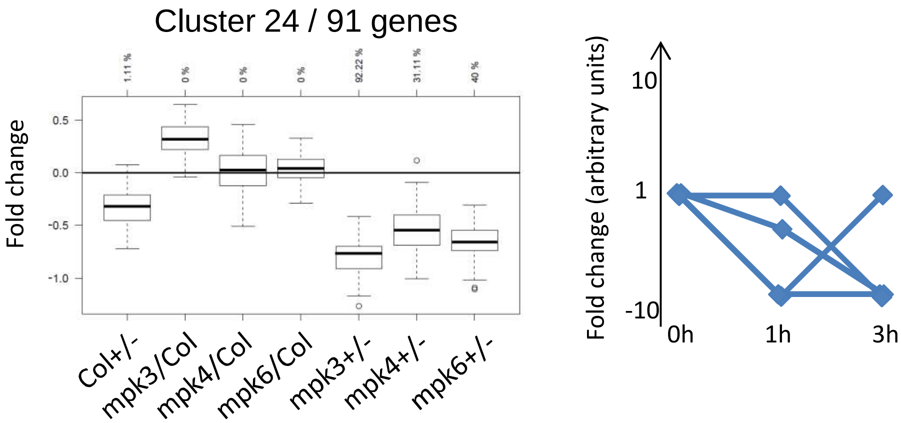

Down-regulated genes (all type of kinetics)
