# Supplementary material for: Functional analysis of Arabidopsis immune-related MAPKs uncovers a role for MPK3 as negative regulator of inducible defences
Source: Genome Biol. 2014 Jun 30;15(6):R87. doi: 10.1186/gb-2014-15-6-r87 (PMC4197828; doi:10.1186/gb-2014-15-6-r87)
Supplement: Additional file 18: Figure S11 — Construction of gene interaction networks. (A) Cytoscape representation of the gene interaction network highlighting the identified regulatory hubs. Zooms into the interaction networks of the regulatory hubs CML9 (B), CPK4 and CPK11 (C), PIF1 (D), HY5 (E). [file gb-2014-15-6-r87-S18.pdf]

Figure S11

Nodes

- TAIR genes not used for the clustering
- Genes differentially expressed but not kept in clustering
- Genes from cluster 10
- Genes from cluster 20
- Genes from cluster 23
- Genes from cluster 19
- Genes from cluster 29
- Genes from cluster 22
- Genes from cluster 18
- Genes from cluster 28
- Genes from cluster 25
- Genes from other clusters

Edges

- TF-target interaction
- Protein-protein interaction

A

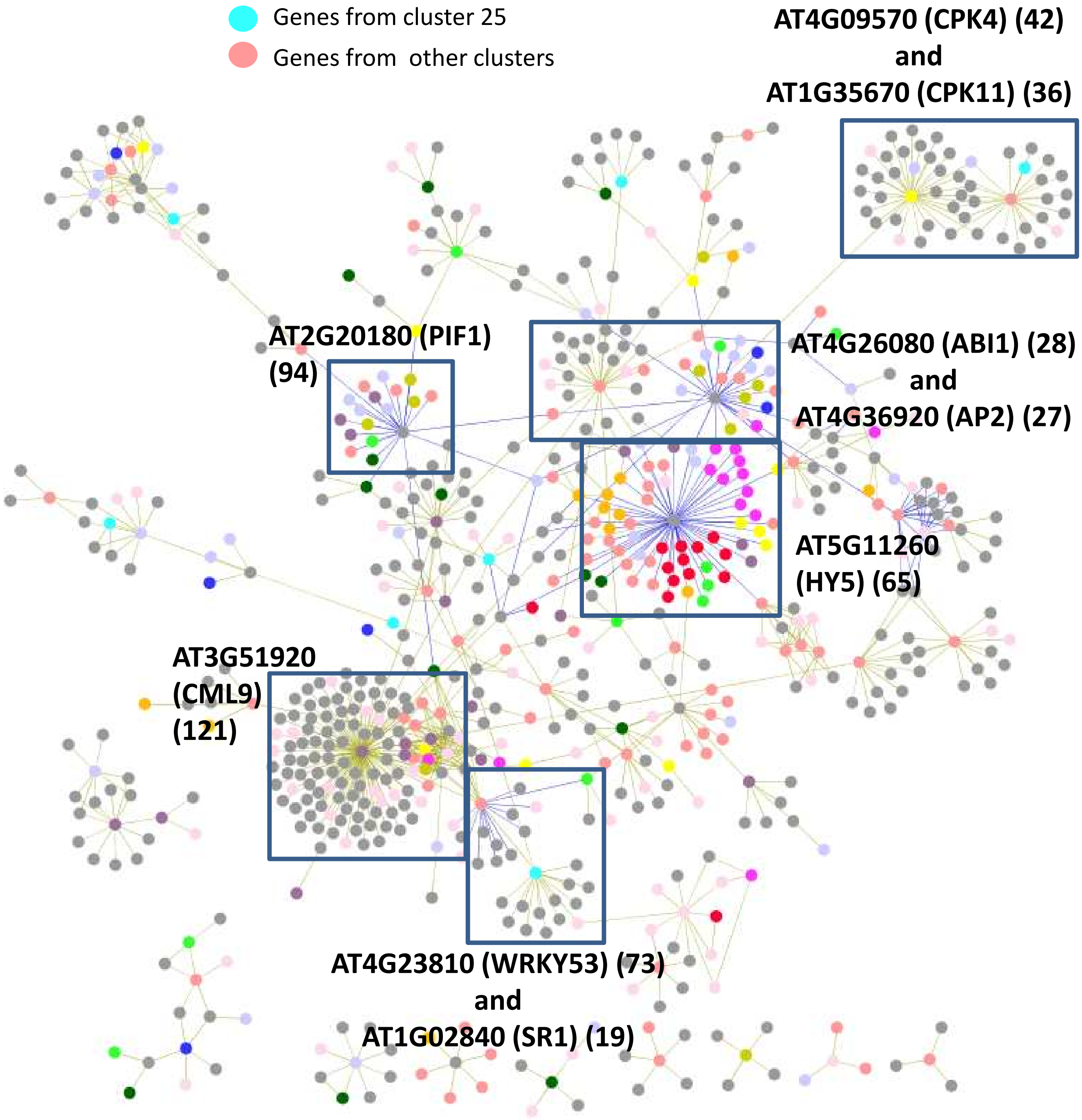

B

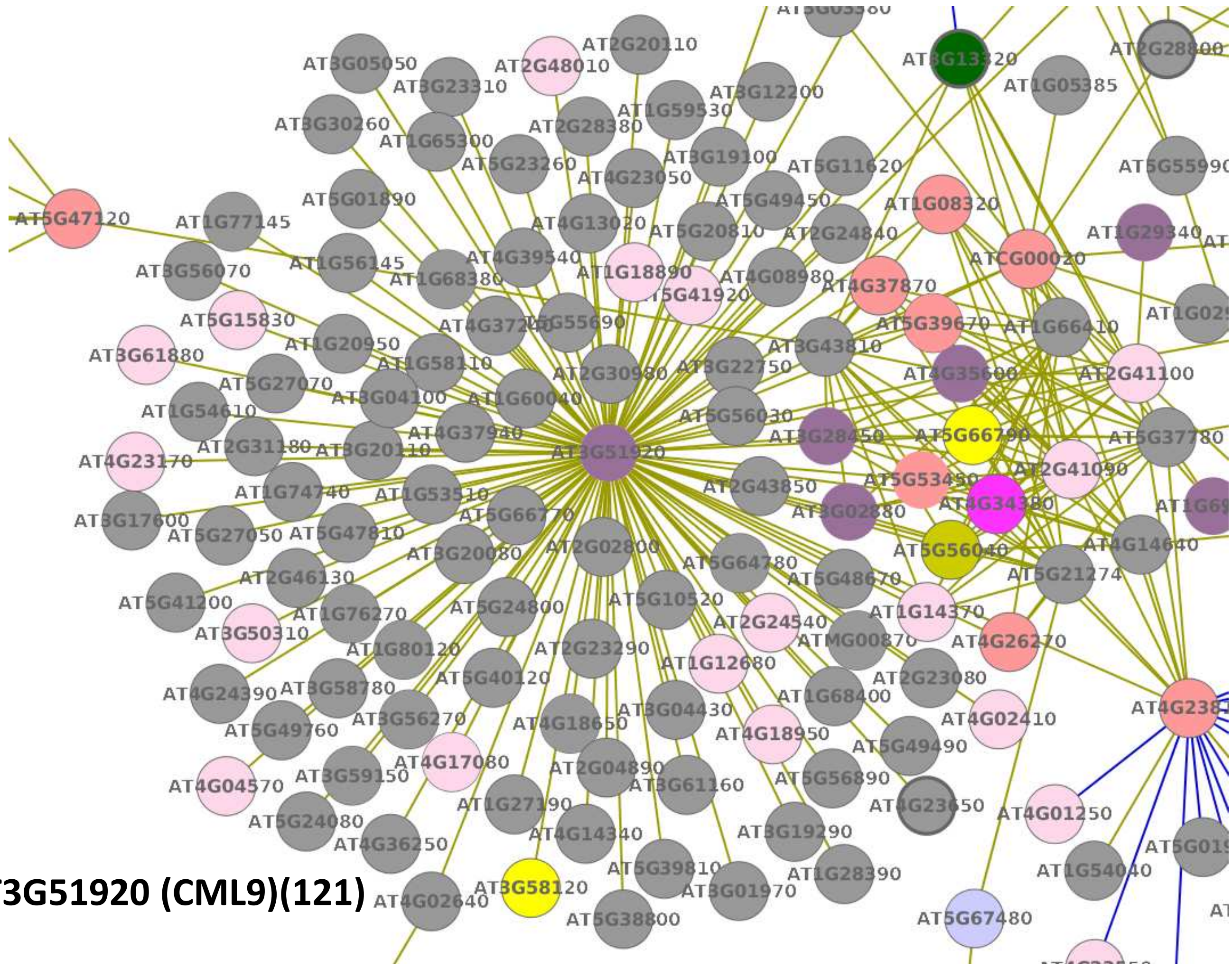

AT3G51920 (CML9)(121)

C

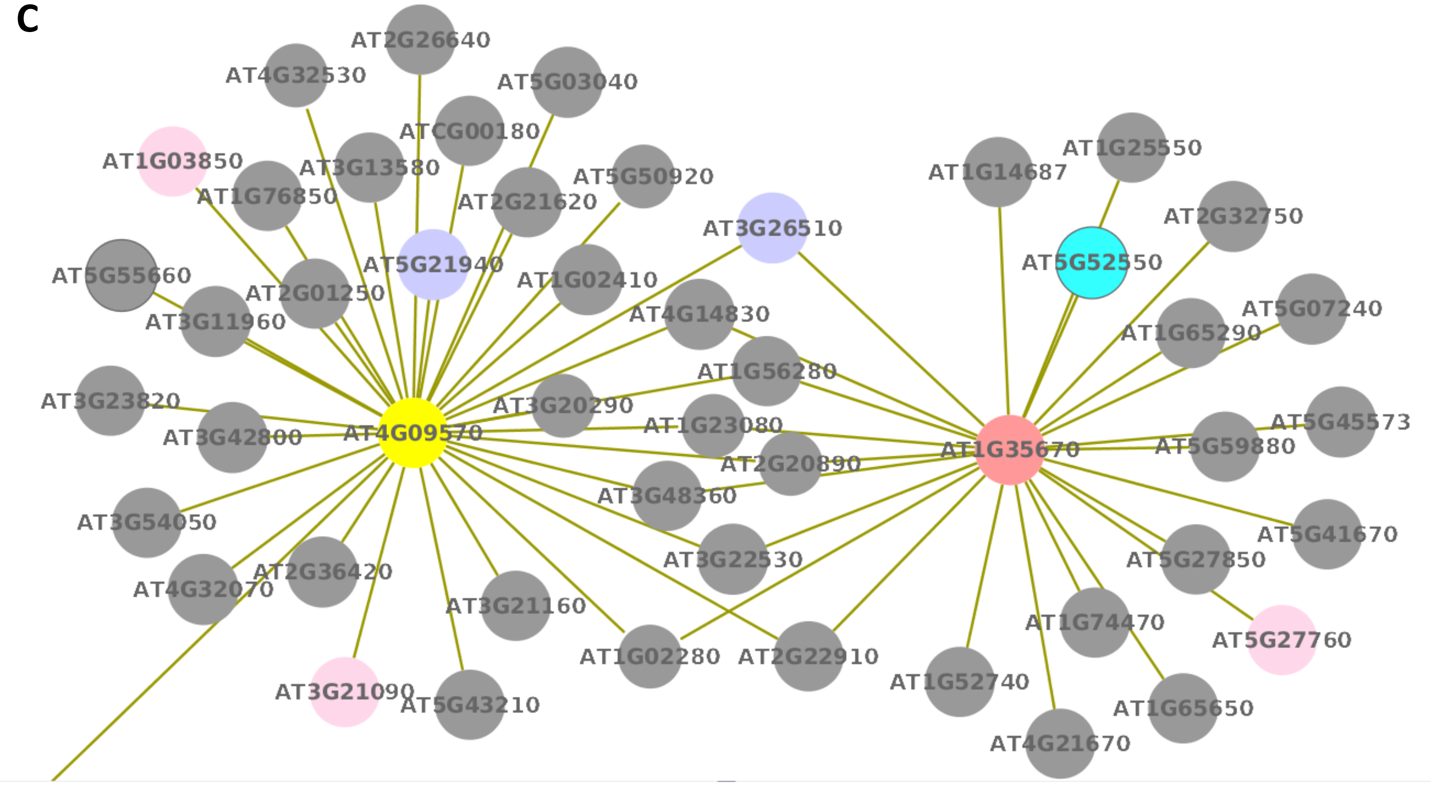

AT4G09570 (CPK4)(42) and AT1G35670 (CPK11)(36)

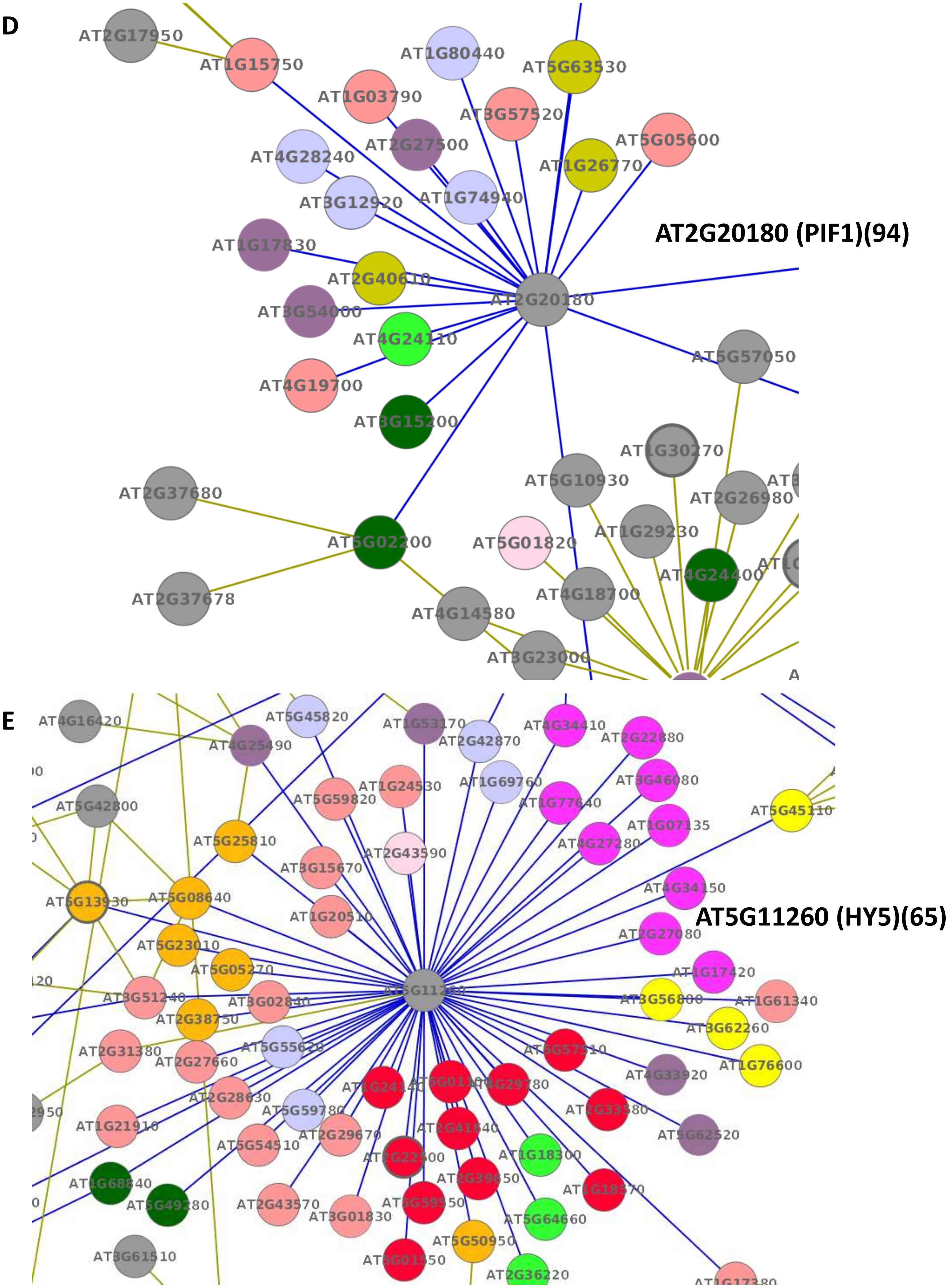
D
AT2G20180 (PIF1)(94)
AT2G20180
AT2G17950
AT1G15750
AT1G80440
AT5G63530
AT1G03790
AT3G57520
AT4G28240
AT2G27500
AT5G05600
AT1G26770
AT3G12920
AT1G74940
AT1G17830
AT2G40610
AT3G54000
AT4G24110
AT4G19700
AT3G15200
AT5G57050
AT1G30270
AT5G10930
AT2G26980
AT2G37680
AT5G02200
AT5G01820
AT1G29230
AT4G24400
AT4G18700
AT2G37678
AT4G14580
AT3G23000
E
AT5G11260 (HY5)(65)
AT4G16420
AT5G45820
AT1G53170
AT4G34410
AT4G25490
AT2G42870
AT2G22880
AT1G24530
AT1G69760
AT3G46080
AT5G42800
AT5G59820
AT1G77640
AT5G45110
AT5G25810
AT2G43590
AT1G07135
AT3G15670
AT4G27280
AT5G13930
AT5G08640
AT4G34150
AT1G20510
AT2G27080
AT5G23010
AT5G05270
AT1G17420
AT3G51240
AT3G02840
AT3G56800
AT1G61340
AT2G38750
AT3G62260
AT2G31380
AT5G55620
AT2G27660
AT5G57510
AT1G76600
AT4G33920
AT2G28630
AT5G59780
AT1G21910
AT2G33580
AT5G62520
AT5G54510
AT2G29670
AT2G41640
AT1G18300
AT1G68840
AT3G49280
AT2G43570
AT3G01830
AT1G18570
AT5G64660
AT3G61510
AT5G50950
AT5G01550
AT2G36220
